# Homophyly/Kinship Model: Naturally Evolving Networks

**DOI:** 10.1038/srep15140

**Published:** 2015-10-19

**Authors:** Angsheng Li, Jiankou Li, Yicheng Pan, Xianchen Yin, Xi Yong

**Affiliations:** 1 State Key Laboratory of Computer Science, Institute of Software, Chinese Academy of Sciences, Beijing, 100190, P. R. China; 2 University of Chinese Academy of Sciences, Beijing, P. R. China; 3 State Key Laboratory of Information Security, Institute of Information Engineering, Chinese Academy of Sciences, Beijing, P. R. China

## Abstract

It has been a challenge to understand the formation and roles of social groups or natural communities in the evolution of species, societies and real world networks. Here, we propose the hypothesis that homophyly/kinship is the intrinsic mechanism of natural communities, introduce the notion of the affinity exponent and propose the homophyly/kinship model of networks. We demonstrate that the networks of our model satisfy a number of topological, probabilistic and combinatorial properties and, in particular, that the robustness and stability of natural communities increase as the affinity exponent increases and that the reciprocity of the networks in our model decreases as the affinity exponent increases. We show that both homophyly/kinship and reciprocity are essential to the emergence of cooperation in evolutionary games and that the homophyly/kinship and reciprocity determined by the appropriate affinity exponent guarantee the emergence of cooperation in evolutionary games, verifying Darwin’s proposal that kinship and reciprocity are the means of individual fitness. We propose the new principle of structure entropy minimisation for detecting natural communities of networks and verify the functional module property and characteristic properties by a healthy tissue cell network, a citation network, some metabolic networks and a protein interaction network.

Real world networks are closely related to human and animal behaviours. According to Darwinian evolution theory^1^, natural selection is the principle of the evolution of species. Based on this principle, we proposed a model of networks characterised by homophyly/kinship to better capture the evolution of high-level real world networks, to understand the principle of natural selection in nature and society, and to understand the mechanisms and laws of natural or true communities in networks, nature and society. Our model demonstrates that social groups or natural communities in nature and society are determined by individual diversity, homophyly/kinship and an affinity exponent. Our results demonstrate that homophyly/kinship and reciprocity are the principles of natural communities in networks, that a balance between homophyly/kinship and reciprocity determined by the affinity exponent realises Darwinian cooperation in a rich-get-richer world in which individuals are selfish (i.e., individuals from natural communities in which most individuals cooperate). We verified that the characteristic properties of networks explored by our model hold for real world networks by examining a gene network, a citation network and some metabolic networks. Our model provides a foundation for both a network theory based on the ideas of biological evolution and a networking approach to biological systems.

Nature and society are supported by numerous networks^2^. Many real world networks follow a power law degree distribution^2^,^3^ and satisfy a small world phenomenon^4^,^5^,^6^.

Community detection is one of the main approaches to understanding the structures of networks^7^,^8^,^9^,^10^,^11^,^12^. Leskovec *et al.*^13^ analysed community structure in large real networks and attempted to find the “best” communities at various sizes, showing that the “best” communities appear to have sizes no more than 100 nodes. Li and Peng^14^ characterised a community as a set, e.g., *S*, of nodes of a graph *G* = (*V, E*), such that the induced subgraph of *S* in *G* is connected, and the conductance of *S* in *G* (or intuitively, the internal density of *S*) is (bounded by a number) inversely proportional to a constant power of the size |*S*| of *S*; that is, 
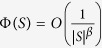
 for a constant *β*. Li and Peng^15^ showed that for each of the classical models, such as 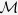, either networks generated by 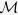 are rich in such communities or are free of the communities.

Kumar *et al.*^16^ found that the WWW graph is rich in bipartite cliques. Based on this, Kumar *et al.*^17^ proposed the copying model by introducing a copying rule in random models to generate power law graphs in which there are rich bipartite cliques interpreted as communities. The idea of copying edges was also used in other models, such as the forest fire model^18^, random walk model, nearest neighbour model^19^ and random-surfer model^20^. Each of these models uses randomness and some local rules including the copying mechanism. The forest fire model generates dense graphs with power law for which the graphs are claimed to have a community structure as a result of the copying operation. The random walk model, the nearest neighbour model and the random-surfer model generate power law graphs in which clustering coefficients are amplified due to the creation of more triangles. The models above demonstrate that power law graphs may be generated by randomness together with some local rules other than the preferential attachment. In addition, the networks generated by models with certain local rules inspired by the copying actions may have interesting subgraphs, such as the connected union of *k*-cliques for *k* ≥ 3 and bipartite cliques, etc. These features together with other properties have been analysed^19^. Albert and Barabási^21^ showed that the frequency of local rules determines the distributions of the generated networks. Palla, Barabási and Vicsek^22^ proposed an algorithm based on clique percolation, allowing us to investigate the time dependence of overlapping communities in large-scale networks. Papadopoulos *et al.*^23^ proposed a static model of networks by introducing similarity based on geometric positions into the preferential attachment scheme.

In his theory of the origin of species, Darwin concluded that animals from ants to people form social groups in which most individuals work for the common good, referred to as Darwinian cooperation, and suggested that natural selection is the controlling principle of the evolution of species^1^. In his book *The Descent of Man*, Darwin suggested that kinship could encourage altruistic behaviour. This concept implies that kinship is the mechanism of social groups in nature. However, the main result of Darwin^1^ is his proposal of individual fitness: individual fitness is key to survival. It has been a longstanding challenge to understand whether Darwinian cooperation and Darwin’s proposal of individual fitness are consistent. How can we achieve both at the same time? By Darwin’s theory, there must be natural community structures in nature and society, corresponding to the true social groups in species and in evolution. Because real world networks are the mathematical representation of complex systems in nature and society, Darwin’s theory implies that there must be natural community structures in real world networks (at least the networks that naturally evolved, in which humans and/or animals are involved). This understanding leads to the following questions: What are the mechanisms of natural communities or social groups in networks? What characteristics and properties do the natural communities of a real world network have? Are there natural communities in real world networks, similar to Darwin’s cooperative social groups in species? What are the natural communities of real networks? Can we achieve Darwinian cooperation in a selfish world?

Li, Li and Pan^24^ proposed a community-finding algorithm based on fitness of networks, a parameter that ensures that all the quality communities have sizes bounded by a polynomial of log *n*, where *n* is the number of nodes of the network, and two prediction algorithms of networks. By using these algorithms on some citation and cooperation networks, we found that real world networks are rich in communities in which most nodes of the same community share common attributes and that the communities have interesting characteristics, such as internal centrality and external de-centrality.

Li, Li and Pan^25^ proposed the notion of structure entropy of a network to quantitatively measure the non-determinism of community structures of the network and a community detection algorithm by minimising the structure entropy of the network. By using the concept of structure entropy of networks and the corresponding algorithm, we have shown that minimisation of structure entropy or equivalently, minimisation of the non-determinism of community structures is both the principle for discovering the natural community structure of a network and the principle of self-organisation of networks in nature and society. This finding establishes the algorithmic principle for identifying the natural community structure of networks.

In this article, we report a local theory of networks, that is, the theory of natural community structures of networks, in which a number of fundamental challenges of networks are resolved.

## Intrinsic Mechanism of Natural Communities

### Homophyly/kinship hypothesis

According to Darwin’s theory^1^, both kinship and reciprocity are the fitness strategies for the evolution of species, which lead to cooperation in social groups and the survival of individuals in the evolution of species.

This theory suggests that kinship and reciprocity could be the mechanisms of natural communities in real world networks, which has been validated by some real world networks^24^. Based on this theory, Li, Li and Pan^24^ proposed the following *homophyly/kinship hypothesis*:

1. Homophyly is an extension of kinship;
2. Homophyly is the intrinsic mechanism of the evolution of real world networks;
3. In real world networks, individuals form natural communities or social groups; and
4. Homophyly/kinship is the mechanism of natural communities in real world networks.

The homophyly/kinship hypothesis not only provides the intrinsic mechanism for exploring the semantics of natural communities but also suggests some syntactic characteristics of natural communities, i.e., the communities generated by homophyly/kinship. Interestingly, according to Li, Li and Pan^25^, minimisation of the (two-dimensional) structure entropy or equivalently, minimisation of the non-determinism of structures of networks, provides the algorithmic principle for identifying the natural communities of networks. Hence, we conclude that homophyly is the intrinsic mechanism and semantic principle of the natural community structure of networks and that, structure entropy minimisation is the algorithmic principle for discovering the natural community structure of networks.

Recently, Song *et al.*^26^ discovered that the potential predictability of human mobility is as high as 93%. This finding indicates that human mobility is highly predictable. The principle behind this discovery could be the homophyly/kinship mechanism.

### Reciprocity hypothesis

Darwin proposed the notion of *reciprocity* to represent the cooperation between individuals in different social groups. He also suggested that reciprocity is a means of individual fitness. He concluded that animals form social groups in which most individuals work for the common good, for which kinship and reciprocity are the mechanisms.

For networks, we proposed the following similar *reciprocity hypothesis*:

- Reciprocity plays an essential role in the formation of natural communities of networks.

The hypothesis suggests the direction of reciprocity study on networks. This direction has not been seriously studied yet and, it would be a new engine for future network theory.

### Individual hypothesis

Before modelling nature evolving and networking, we needed to understand the basic properties of the individuals of a real world network. We proposed the following hypothesis to capture the basic properties of individuals in nature and society.

*Individual hypothesis*:

1. Every individual is a local existence;
2. Every individual is observably different from others; and
3. Every individual plays a role.

In animals, every individual eats for survival, and different individuals eat different things. In nature and society, every individual has its own characteristics, plays its own roles and has its own rights, simply due to the birth of the individual.

The individual hypothesis represents the existence, roles and rights of an individual in nature and society.

## Principles of Nature Evolving

### Homophyly/kinship model

We built our model based on the homophyly/kinship hypothesis and individual hypothesis, as described above. Note that the reciprocity hypothesis will be automatically guaranteed by the model.

However, the proposed individual hypothesis and homophyly/kinship hypothesis cannot completely determine a network, a society or a species automatically. For this, we introduced the notion of the *affinity exponent* to quantitatively measure the force of the homophyly/kinship of a species, a society or a network. The notion of affinity is different from but matches homophyly/kinship in an interesting way; the former represents the intention of a community to accept a new member, and the latter means that individuals in a community are strongly linked together.

Based on this information, we proposed our model using our homophyly/kinship hypothesis and by introducing the notion of affinity exponent, based on our individual hypothesis. The model, referred to as the *homophyly/kinship model*, proceeds as follows.

#### Homophyly/kinship model

Given affinity exponent *a* ≥ 0 and natural number *d*:

1. Let *G*_*d*_ be an initial *d*-regular graph in which each node is associated with a distinct colour and is called a seed.(The initial graph could be an arbitrarily given graph, which does not change the results of the model.)For *i* > *d*, let *G*_*i*−1_ be the graph constructed at the end of step *i* − 1, and let 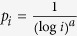.
2. At step *i*, we create a new node *v*.
3. (Preferential attachment) With probability *p*_*i*_, *v* chooses a new colour, in which case
  - we call *v* a seed, and
  - create *d* edges from *v* to nodes in *G*_*i*−1_, chosen with probability proportional to the degrees in *G*_*i*−1_.
4. (Homophyly/kinship) Otherwise, then *v* chooses an old colour, in which case
  - *v* chooses randomly and uniformly an old colour as its own colour. and
  - creates *d* edges from *v* to nodes of the same colour in *G*_*i*−1_, chosen with a probability proportional to the degrees in *G*_*i*−1_.

The motivation of the selection of 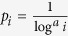 in our model is that this is the unique choice that is mathematically correct. The reasons are as follows: (1) *p*_*i*_ must be a function of *i*; otherwise, the model will be either trivial or static (if it is a function of *n*, the number of nodes); and (2) intuitively, for a network of *n* nodes, the basic module of the network should be remarkably smaller than *n* quantitatively, which should be bounded by a polynomial of log *n*. If we assume (1) and (2), the only choice of *p*_*i*_ is 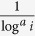.

In the literature, the model of networks that is closely related to ours is that in Papadopoulos *et al.*^23^, which introduces similarity (a form of homophyly) into the preferential attachment. The model generates networks as follows: (1) randomly sample *n* nodes in a plane, (2) define a similarity by the relative positions on nodes in the plane, and (3) create links by both popularity (a form of preferential attachment) and similarity. The model generates networks with both power law and community structures. However, the model is static and geometric. More importantly, the definition of similarity by relative positions in the model reflects a form of “social selection” and “peer influence” in the formation of social groups. Certainly, “social selection” and “peer influence”, if well defined, are factors in the formation of social groups. However, all of these factors are extrinsic causes of the formation of social groups. Therefore, Papadopoulos *et al.* explain some of the extrinsic factors of social groups. Our homophyly/kinship model focuses on the intrinsic mechanism of the formation of social groups; that is, an individual has an attribute and an individual must play its own roles in its social groups and in the network. In particular, every individual wants to survive. In addition, our homophyly/kinship is dynamic and discrete. Of course, it is interesting to study the extrinsic factors of the formation of natural communities in networks; among all the possible extrinsic factors, game could be the key, and it has never been addressed in the literature. This understanding suggests a new direction to study the role of games in the formation of natural communities in networks, nature and society.

The most notable research that shares some of the ideas of our model is the work of Guimerá and Amaral^27^. They assumed that individuals play roles on their own, in their communities, and in whole networks. Our model realises this idea by the individual hypothesis. This paper describes a community-finding algorithm based on modularity with simulated annealing on some metabolic networks to find the functional modules of the networks. This paper divides all the nodes into 7 different roles based on the patterns of the found communities. In particular, it was shown that for some networks, 80% of nodes have links only to nodes within their own communities.

Another interesting paper was authored by Palla, Barabási and Vicsek^22^, who analysed the properties of time evolution of some algorithmic communities. However, this approach is completely different from the evolution of our model.

Finally, we emphasise that our model focuses on the intrinsic mechanism of natural communities of networks and explores the laws of the mechanism. The model constructs networks dynamically with both homophyly/kinship and preferential attachment as its mechanisms.

### Homophyly/kinship principle

We showed that networks of our homophyly/kinship model satisfy a *homophyly/kinship principle*.

Given the affinity exponent *a* ≥ 0 and natural number *d*, let *G* = (*V, E*) be a network of our homophyly/kinship model. We say that a maximal homochromatic set of *V* is a *natural community* of *G*. The homophyly/kinship principle consists of the following laws and properties (full proofs of the principle will be referred to in the supplementary materials of the paper):

1. (*Self-organising law, holographic law, and power law*^3^) The degrees of the induced subgraph of a natural community, the degrees of the natural community and the degrees of the whole network all follow a power law with the same power exponent.

#### Remark

Self-organisation is a basic phenomenon of networks.

1. above states the results of self-organisation behaviours in networks. According to our homophyly/kinship model, homophyly/kinship is the mechanism of self-organisation. According to the theory of structure entropy^25^, minimisation of the structure entropy or the non-determinism of structures is the principle of self-organisation behaviours in networks. These results together demonstrate that homophyly/kinship, minimisation of non-determinism of structures and holographic law together with a power law are the intrinsic cause, principle and results of self-organisation behaviours in networks, respectively.
2. (*The small community phenomenon*^14^,^15^) A natural community contains at most *O*(In^*a*+1^*n*) individuals. This statement follows from both a counting argument and a probabilistic argument and indicates that a natural community is always small.
3. (*Local communication law*) The diameter of a natural community is *O*(log log *n*), where *n* is the number of nodes in *G*.This statement follows from (2) and the proof of small diameter of graphs by the preferential attachment^3^.
4. (*Small diameter property*^4^,^5^,^6^) The diameter of *G* is bounded by 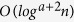.This statement follows from (2) and the fact that the diameter of a graph by the preferential attachment is *O*(log *n*).
5. (*Natural community law*) Let *X* be a maximal homochromatic set. Then, the induced subgraph *G*_*X*_ of *X* is connected, and the conductance of *X*, written by Φ(*X*), is 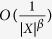 for some constant *β*.Institutively, this result indicates that the density of internal links of a natural community is bounded by a number inversely proportional to a constant power of the size of the community, which is fundamental to understanding the natural communities.
6. (*Degree priority law*) Given a node *v* ∈ *V*, we defined the *length of degrees of v* to be the number of colours associated with all the neighbours of *v*, written by *l*(*v*). For *j* ≤ *l*(*v*), we defined the *j*-th degree of *v* to be the *j*-th largest number of edges of the form (*v, u*), such that the *u*’s here share the same colour, denoted by *d*_*j*_(*v*). The degree of *v, d*(*v*), is defined as the number of edges incident to node *v*.Then, almost surely, or with probability 1 − *o*(1), the degree priority of a node *v* in *V* satisfies the following properties: (i) (First degree property) The first degree of *v, d*_1_(*v*) is the number of edges from *v* to nodes of the same colour as *v*. (ii) (Second degree property) The second degree of *v* is bounded by a constant, i.e., *d*_2_(*v*) ≤ *O*(1). (iii) (The length of degrees) The length of degrees of *v* is bounded by *O*(log *n*). (iv) If *v* is a seed node, the first degree of *v, d*_1_(*v*), is at least 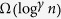 for 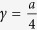.This statement indicates that the degree of a node is contributed mainly by nodes of its own natural community. This law allows us to analyse the patterns of a natural community to link to nodes outside the community.
7. (*King node property*) With a high probability, for a natural community *X* and its seed node *x*_0_, the degree of *x*_0_ is significantly larger than that of its neighbours in its own community.This is a statistical law, for which the reasons are as follows: When the seed *x*_0_ is created, we create *d* edges from *x*_0_ to the existing nodes. At the time step at which the second node *x*_1_, say, of the natural community *X*, is born, the degree of *x*_1_ is *d*, and simultaneously, the degree of *x*_0_, is at least 2*d*. This approach gives a significant initial advantage of *x*_0_ over *x*_1_. By the preferential attachment of the construction of *X*, the initial advantage of *x*_0_ is probably maintained or strengthened during the construction of the network. The king node property implies that every social group of animals or human societies has a leader or a leadership, similar to a colony of honey bees, ants, or humans, thus truthfully reflecting the real world.
8. (*Inclusion-exclusion property*) With a high probability, for two distinct natural communities *X* and *Y*, the number of edges between *X* and *Y* is *e*(*X, Y*) = *O*(1), i.e., some constant independent of the sizes of the communities. [For real world networks, *e*(*X, Y*) may not be constant, but it must be remarkably smaller than the sizes of *X* and *Y*. Theoretically, *e*(*X, Y*) could be exponentially smaller than the sizes of *X* and *Y*].This statement indicates that two distinct communities have few connections. This property ensures that the natural communities are relatively independent from each other. Otherwise, two communities may easily merge into one community.
9. (*Reciprocity*) Let *X* be a natural community of *G* and *x*_0_ be the seed of *X*. We defined the *reciprocity of x*_0_ *and X* to be the number of edges from *x*_0_ and *X* to nodes outside *X*, written by recip(*x*_0_) and recip(*X*), respectively. Then, with a high probability, both recip(*x*_0_) and recip(*X*) are significantly large, meaning that both the seed of the community and the natural community have multiple links to nodes outside the community. This property would be important for the survival of the community according to Darwin’s theory.

This property says that a natural community has good connections with the nodes outside the community, which ensures that a community has a chance to gain benefits from the nodes outside the community.

(Remark. To understand (8) and (9), let us consider the example of international trade. In this example, a community is a country. (9) shows that for a country *X*, although international trade contributes only a small part of *X*’s economy, without this small part of international trade, the country will be isolated and will fail. (8) shows that if all the international trade of *X* are with just one country *Y*, the international trade of *X* is unhealthy, in which case there is a risk that *X* may be easily controlled by *Y*. In fact, by (8), we know that it is the best strategy for a country *X* to have its international trade evenly distributed among different countries.)

The properties above explore the mathematical principles of homophyly/kinship in networks.

## Characteristic Properties of Natural Communities

For the appropriate affinity exponent *a*, let *G* be a network of our homophyly/kinship model with affinity exponent *a*. By the homophyly/kinship principle above, we proposed the following *characteristic properties of natural communities*: (1) (*Interpretability*) Most nodes of a natural community share a short list of common attributes (colour). (2) (*Robustness*) Most nodes in a natural community have neighbours only within their own community. (3) (*Stability*) For every node *v*, the degree of *v* is largely contributed by nodes of *v*’s own natural community. (4) (*Leadership*) A natural community has a leading node whose degree is significantly larger than that of its neighbours in its own community. (5) (*Internal centrality*) A natural community has a few nodes that dominate the community. (6) (*External de-centrality*) The neighbours of a natural community that are outside the community are homogenously distributed in different communities. (7) (*Reciprocity*) A natural community has a significantly large number of edges from the nodes of the community to nodes outside the community. (8) (*Independency and inclusiveness*) The number of edges between two different natural communities is a small number; in fact, the number is usually as small as a constant.

For a network of our model with appropriate the affinity exponent *a*, the properties (1)–(8) are the results of the homophyly/kinship principle. This also means that the properties (1)–(8) are principally determined by the affinity exponent *a*, together with slight variations by *d* as usual. This implication is one of the most interesting discoveries. For example, we showed that the curves of robustness and stability increase as the affinity exponent *a* increases and that the curve of the reciprocity decreases as the affinity exponent *a* increases. This fining indicates that if we want a network to have quality robustness, stability and reciprocity simultaneously, we must choose an appropriate affinity exponent *a*, which must not be too small and not too large. We showed that the selection of such an *a* is essential to the emergence of cooperation in evolutionary prisoner’s dilemma games in networks of our model. This finding also implies that a well-evolved network in the real world certainly has high quality properties of robustness, stability and reciprocity, which must be determined by an appropriate affinity exponent *a*.

Clearly, properties (1)–(8) above not only capture the characteristics of natural communities in networks but also determine the roles of the communities in the networks and the roles of the networks. The properties provide a theoretical resource for analysing the natural or true communities, the roles and functions of the communities in real world networks, and the network properties of real networks in nature and society.

We verified that networks of our model with the appropriate affinity exponent *a* do satisfy the properties (1)–(8) of natural communities.

### Robustness of natural communities

Let *X* be a natural community of *G*. By definition, (most) nodes in a natural community share common attributes. In the real world, a natural community would be a basic unit, or a functional module of a network, a society or a species. This statement implies that there must be some *intrinsic force* to bring the individuals of a natural community together. To capture this phenomenon, we defined the notion of *robustness* of the natural communities of a network.

Given a community *X*, we defined the *robustness of X* as follows:


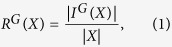


where *I*^*G*^(*X*) is the set of nodes *x* ∈ *X*, such that all the neighbours of *x* are in *X*.

**Figure 1:**
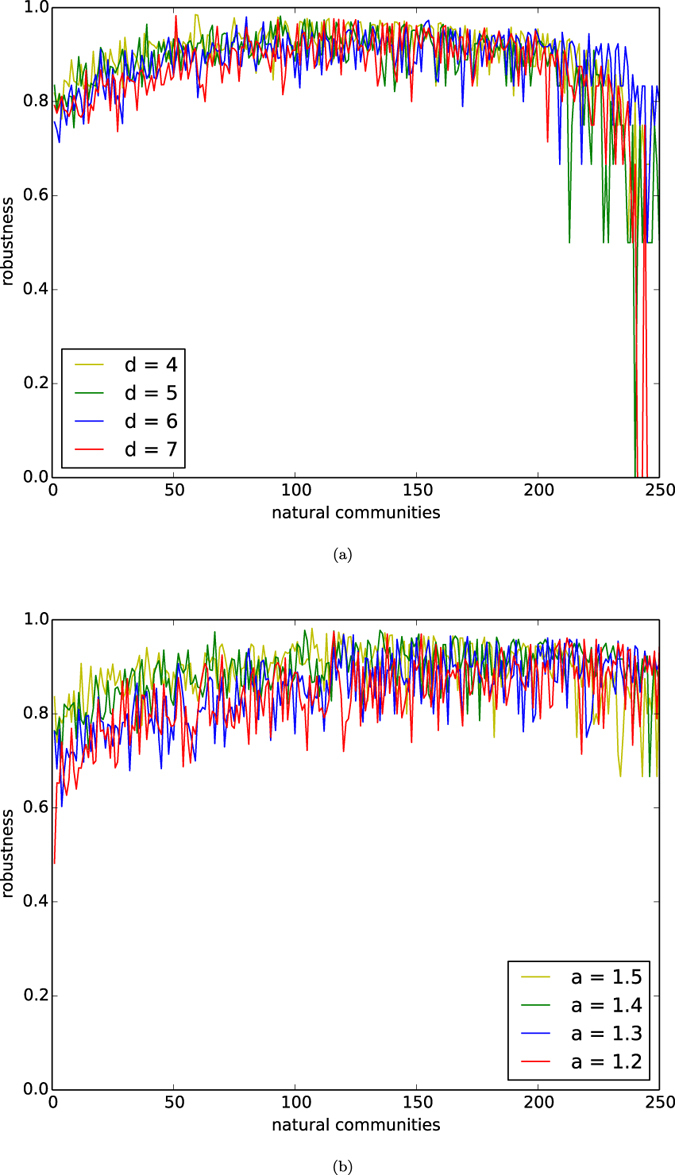
Robustness. (**a**) Robustness of natural communities of the networks of our homophyly/kinship model with *n* = 10,000, the affinity exponent *a* = 1.5, and *d* = 4, 5, 6, 7. (**b**) Robustness of natural communities of the networks generated by our homophyly/kinship model with *n* = 10,000, *d* = 5 and the affinity exponent *a* = 1.2, 1.3, 1.4, 1.5.

In Fig. 1(a), we depict the curves of robustness of all the natural communities by the ordering of the birth of the communities in the networks of our homophyly model with affinity exponent *a* = 1.5, *n* = 10,000, and *d* = 4, 5, 6, 7. By observing Fig. 1(a), we demonstrate the following results:

1. For each of the *d*’s from 4 to 7, the robustness function *R*(*X*) is higher than 0.6 or even higher than 0.8 for all community *X*’s, except for the few most recently created communities.
2. For each *d*, the robustness function *R*(*X*) for the few earliest born communities is slightly less than that of the majority of the natural communities.
3. Statistically, the robustness curve for the networks with *d* = 7 is slightly lower than that for the network with *d* = 4.
4. By (3), we have that, statistically, the robustness of natural communities slightly decreases as *d* significantly increases.

By (1) and (2), most natural communities are robust in the sense that the curve of the robustness function is higher than 0.6, except for the few newly born communities.

In Fig. 1(b), we depict the curves of the robustness function *R*^*G*^(*X*) for all the natural community *X*’s by the ordering of the birth of the communities, for four networks *G*’s with *n* = 10,000, *d* = 5, and *a* = 1.2, 1.3, 1.4, 1.5. By observing Fig. 1(b), we demonstrate the following results:

1. For each of the affinity exponent *a*’s from 1.2 to 1.5, the robustness function *R*^*G*^(*X*) is higher than 0.6 or even higher than 0.8 for all community *X*’s, except for the few most recently created communities.
2. For each *a*, the robustness function *R*(*X*) for the few earliest born communities is slightly less than that of the majority of the natural communities.
3. Statistically, the robustness curve for the networks with *a* = 1.2 is slightly lower than that for the network with *a* = 1.5.
4. By (3), the robustness of natural communities increases as the affinity exponent *a* increases.

By (1) and (2), most natural communities are robust in the sense that the robustness function is higher than 0.6, except for the few newly created communities.

The experiments in Fig. 1(a,b) show that for fixed number of nodes *n*, the robustness of natural communities of the networks of our homophyly/kinship model slightly decreases as *d* significantly increases, and slightly increases as the affinity exponent *a* increases. The result implies that natural communities of networks are robust, and the robustness of natural communities is determined by the affinity exponent of the networks.

As mentioned before, one of the main discoveries in Guimerá and Amaral^27^ was that for some metabolic networks, there are functional modules such that, 80% of nodes link to nodes only within their own modules. The robustness of networks of our homophyly/kinship model aligns with the discovery by Guimerá and Amaral^27^. Our results also imply that the metabolic networks may correspond to the appropriate affinity exponent *a*. (We will see later that robustness is only one aspect of network characteristics because maximal robustness may be achieved by trivial classification consisting of only a few, e.g., 2 or 3, communities. For this reason, we are concerned with the natural communities, instead of those that are optimal for a specific measure.)

### Stability of natural communities

Let *G* = (*V, E*) be a network generated by the homophyly/kinship model. Suppose that 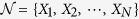 is the set of all natural communities of *G* listed by the order of the birth of the seeds of the natural communities.

For a natural community *X* = *X*_*i*_ for some *i* and for a node *x* ∈ *X*, we define the *stability of x in X* by


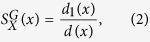


where *d*_1_(*x*) is the number of edges of the form (*x, y*) for *y* ∈ *X*, and *d*(*x*) is the degree of *x* in *G*.

The notion of stability is again to understand the reason why a group of individuals form a natural community in the sense that the main payoffs of the majority of individuals of the group can be obtained through the links within the group.

**Figure 2:**
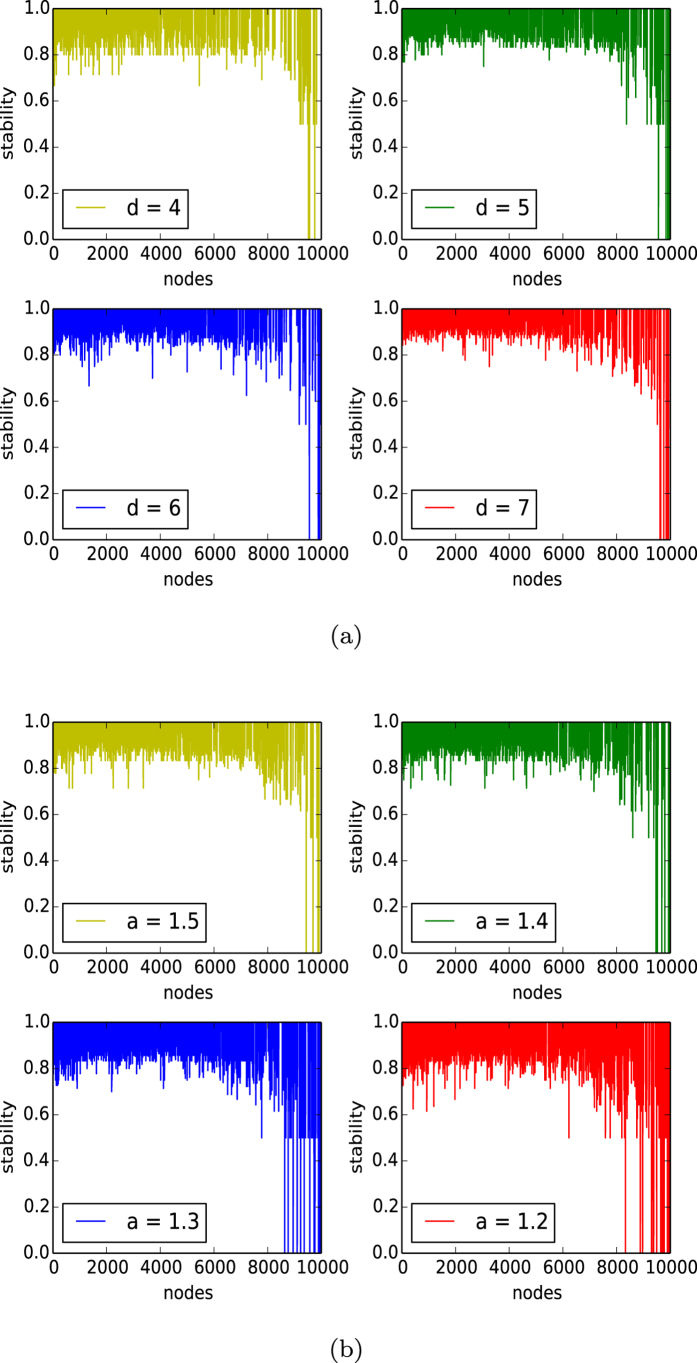
Stability. (**a**) Stability of individuals of the networks of the homophyly/kinship model with *n* = 10,000, and affinity exponent *a* = 1.5, in which the four panels (**a**–**d**) are the curves of the networks with *d* = 4, 5, 6, 7, respectively. (**b**) Stability of individuals of networks of the homophyly/kinship model with *n* = 10,000, and *d* = 5, in which the four panels (**a**–**d**) are the curves for the networks with the affinity exponent *a* = 1.2, 1.3, 1.4, 1.5, respectively.

In the four panels of Fig. 2(a), we depict the distribution of the stabilities *S*(*x*) for all the node *x*’s in networks of the homophyly/kinship model with *n* = 10,000, affinity *a* = 1.5, and *d* = 4, 5, 6, 7. By observing Fig. 2(a), we demonstrate the following results:

1. For each *d*, the stabilities of almost all nodes are larger than 0.8, except for the few newly created nodes.
2. The curves of stability distributions of the networks with different *d*’s are all the same.(
  1. shows that all nodes are stable, except for the few newly born nodes. (2) shows that the stability of networks of our model is independent of the choices of *d*.

In Fig. 2(b), we depict the curves of the stabilities of the networks of our homophyly/kinship model with *n* = 10,000, *d* = 5, and affinity exponent *a* = 1.2, 1.3, 1.4, 1.5. By observing Fig. 2(b), we have the following results:

1. For each affinity exponent *a*, the stabilities of almost all nodes are larger than 0.8, except for the few newly created nodes.
2. By comparing the figures in panels (a) and (b) of Fig. 2(b), we know that the curves of stability distributions of the networks increase as the affinity exponent *a* increases.

By the experiments shown in Fig. 2(a,b), we demonstrate that the curve of stabilities of the networks of our model is independent of the choice of *d* and that the curve of stabilities of the networks of our model increases as the affinity exponent *a* increases. The results imply that the stability of networks of our model is determined by the affinity exponent *a*.

### Leadership of natural communities

Let *G* be a network of our model. For a natural community *X*, let *x*_0_ be the seed of *X*. We define the *leadership of X* by


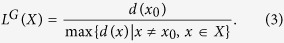


**Figure 3:**
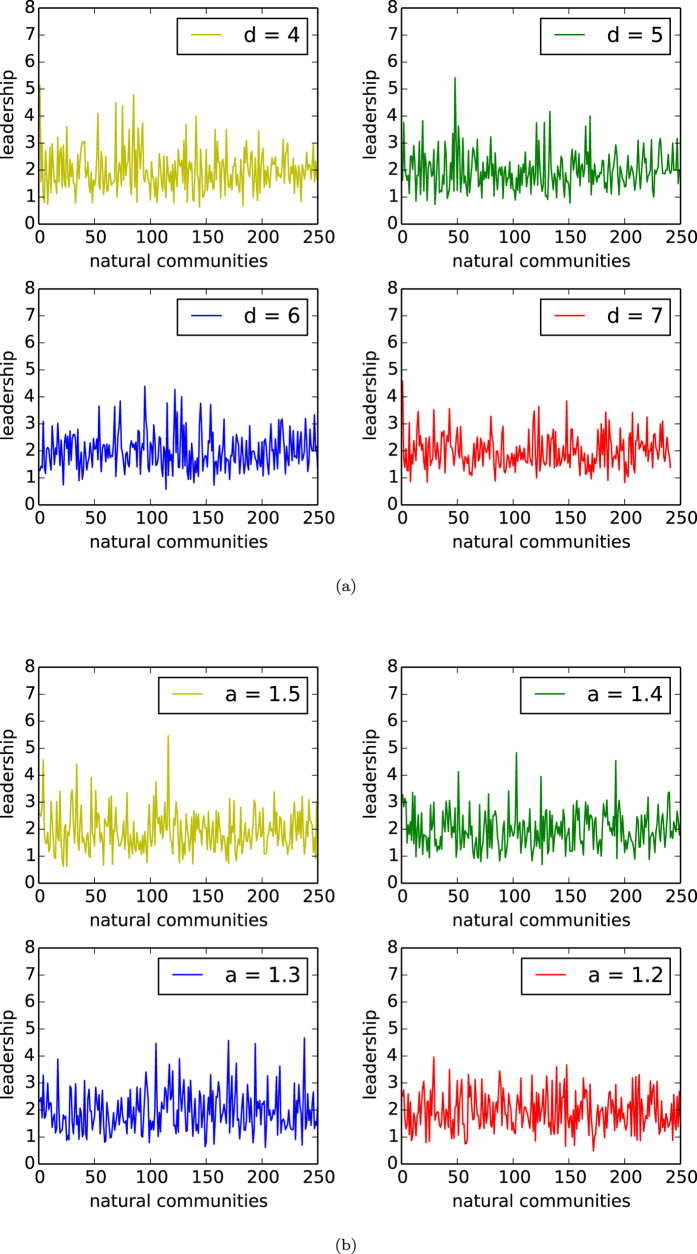
Leadership. (**a**) Leadership of natural communities of the networks with *n* = 10,000, the affinity exponent *a* = 1.5 of our model, in which the four panels (**a**–**d**) are the curves for *d* = 4, 5, 6, 7, respectively. (**b**) Leadership of natural communities of networks with *n* = 10,000, *d* = 5 of our model, in which the four panels (**a**–**d**) are the curves for the affinity exponent *a* = 1.2, 1.3, 1.4, 1.5, respectively.

In Fig. 3(a), we depict the curves of the distributions of leadership of natural communities of networks of our model with *n* = 10,000, affinity *a* = 1.5, and *d* = 4, 5, 6, 7. In Fig. 3(b), we depict the distributions of leadership of all the natural communities of networks of our homophyly/kinship model with *n* = 10,000, *d* = 5, and affinity exponent *a* = 1.2, 1.3, 1.4, 1.5.

By observing Fig. 3(a,b), we demonstrate the following results:

1. In each network, the leadership of almost all the natural communities is significantly larger than 1.
2. In each network, the expectation of the leaderships of all the natural communities is 2.

The results show that in every such network, almost surely, the seed node is the highest degree node in its natural community and that with high probability, the degree of a seed node is significantly larger than any non-seed nodes of its own community. This phenomenon is similar to many species in nature. For example, a colony of honey bees has a queen bee, which is larger than the other bees in its colony. A similar phenomenon also occurs for other animals. (This notion is interesting because it may better reflect the natural communities of animals.)

Generally, the experiments here imply that different individuals in a natural community may have different properties and play different roles. In particular, a natural community may have a few important individuals, referred to as *hubs*. The hubs of a natural community may lead the community in some common interests, and may play more important roles in linking to individuals outside their own community. In network applications, identification of the different roles of different types of individuals is extremely important. For instance, a cell type of cancer consists of a group of cells, in which each cell may play a different role. In this case, to further distinguish the different roles of cells in the type would be very important for diagnosis and therapy of the tumour. Our results imply that the distinct roles of individuals in a natural community may be determined by the structure or pattern of the community.

### Internal centrality of natural communities

Let *X* be a natural community. We find a dominating set *D* of the induced subgraph *G*_*X*_. We define the *dominating ratio of X* to be 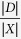.

The notion of the dominating ratio is to identify the core of a natural community. Usually, the natural communities are heterogeneous. Therefore, the dominating ratios are remarkably smaller than the communities.

**Figure 4:**
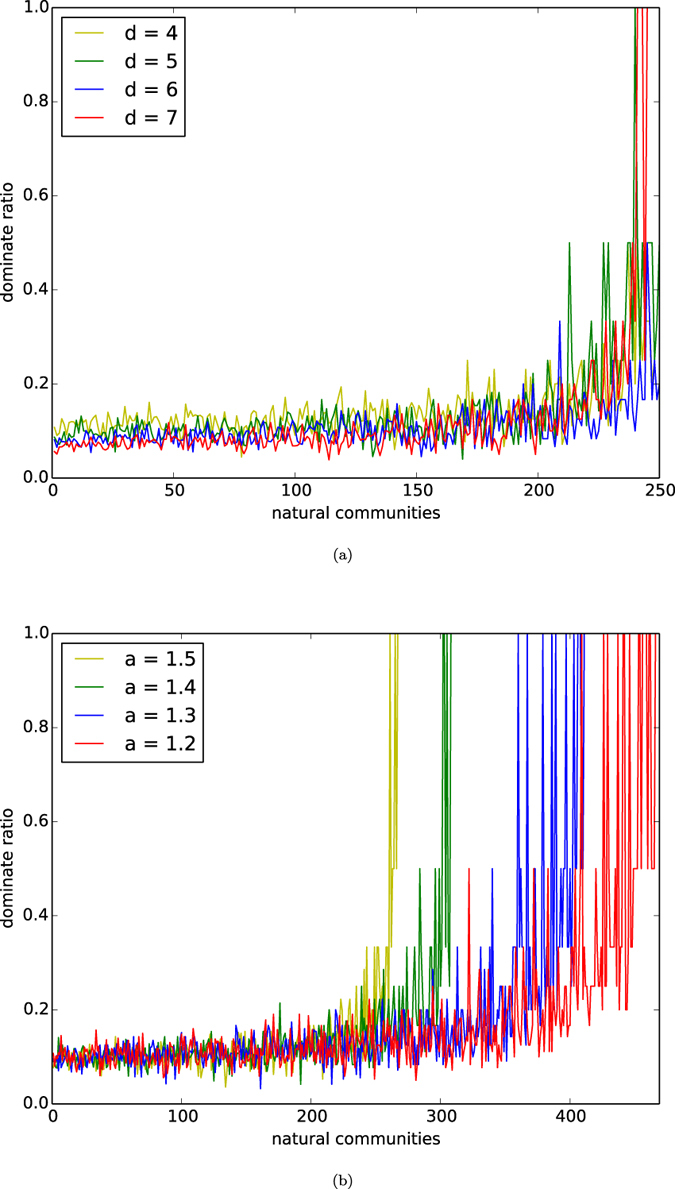
Dominating sets. (**a**) Dominating ratios of natural communities of the networks of our model with *n* = 10,000, the affinity exponent *a* = 1.5 and *d* = 4, 5, 6, 7. (**b**) Dominating ratios of natural communities of the networks with *n* = 10,000, *d* = 5, and the affinity exponent *a* = 1.2, 1.3, 1.4, 1.5.

In Fig. 4(a), we depict the distributions of the dominating ratios of natural communities of networks of the homophyly/kinship model with *n* = 10,000, affinity exponent *a* = 1.5, and *d* = 4, 5, 6, 7. In Fig. 4(b), we depict the distributions of dominating ratios of natural communities of networks of our model with *n* = 10,000, *d* = 5, and affinity exponents *a* = 1.2, 1.3, 1.4, 1.5.

By observing Fig. 4(a,b), we demonstrate:

1. For each *d*, the early born communities are well evolved, each of which has a small dominating set.
2. For each affinity exponent *a*, the early born communities are well-evolved, each of which has a small dominating set.

The results in (1) and (2) above further show that a natural community has an internal centrality, and that a natural community has a few important nodes dominating the whole community, unless the community has not been well-evolved yet.

This result may be used in a recommendation system. For instance, when we find a community *X*, we recommend a dominating set *D* of *X*. The dominating set *D* of *X* is much smaller than the community *X*, from which we already have much knowledge of *X*, and from which we can easily access *X*.

### Reciprocity of natural communities

Given a network *G* of our model, let *X* be a natural community of *G*. We define the *reciprocity of X in G* as follows:


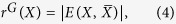


where *E*(*X, Y*) is the set of edges between *X* and *Y*, and 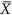 is the complement of *X*.

Reciprocity of a natural community *X* ensures that nodes in *X* are well-connected to nodes outside of *X*, and that, nodes in *X* may gain extra interests from nodes outside *X* (in games, for instance).

**Figure 5:**
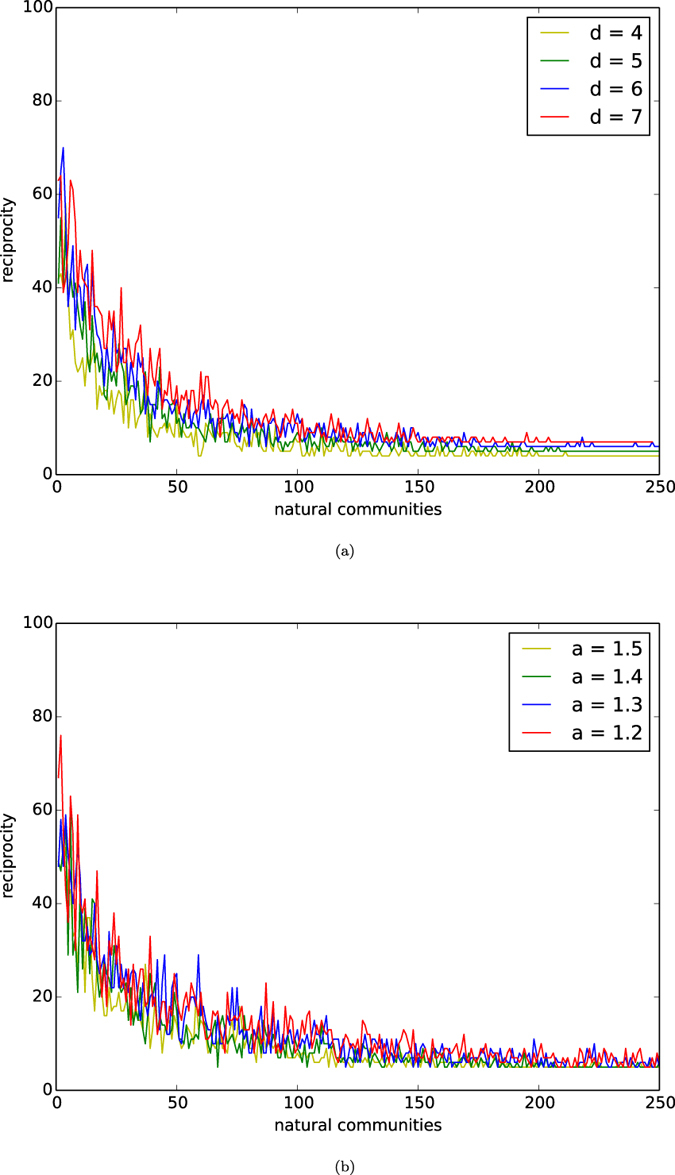
Reciprocity. (**a**) The curves are the distributions of reciprocity or networks of the model with *n* = 10,000, affinity exponent *a* = 1.5, and *d* = 4, 5, 6, 7. (**b**) The curves are the distributions of reciprocity or networks of the model with *n* = 10,000, *d* = 5, and affinity exponents *a* = 1.2, 1.3, 1.4, 1.5.

In Fig. 5(a), we depict the distributions of reciprocities of natural communities of networks of the homophyly/kinship model with *n* = 10,000, affinity exponent *a* = 1.5, and *d* = 4, 5, 6, 7.

In Fig. 5(b), we depict the distributions of reciprocities of natural communities of networks of our model with *n* = 10,000, *d* = 5, and affinity exponents *a* = 1.2, 1.3, 1.4, 1.5.

By observing Fig. 5(a,b), we have the following results:

1. Stochastically, for a network *G* with *N* natural communities, the reciprocity of the *i*-th community *X*_*i*_ is approximately equal to 
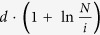
.
2. The affinity exponent *a* stochastically determines the number of natural communities of a network, *N* say, for which we have that 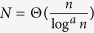, where *n* is the number of nodes of the network *G*.
3. By (1) and (2), reciprocity increases as *d* increases.
4. By (1) and (2), reciprocity decreases as the affinity exponent *a* increases.

By (1), for most natural community *X*_*i*_’s, the reciprocities are significantly large. (2) gives an estimation of the number of natural communities determined by the affinity exponent. (3) and (4) indicate that reciprocities of the natural communities are determined by both *d* and the affinity exponent *a*. Recall the results of robustness and stability of natural communities, and we have the following interesting results:

1. Robustness decreases as *d* increases,
2. Robustness and stability increase as *a* increases,
3. Reciprocity increases as *d* increases, and
4. Reciprocity decreases as *a* increases.

The results show that robustness/stability and reciprocity are contradictory, that for given *d*, robustness, stability and reciprocity are determined by the affinity exponent *a*, and that the affinity exponent *a* may determine a trade-off or balance between robustness/stability and reciprocity.

This result is a surprising discovery; we may interpret robustness and stability as a homophyly/kinship because both robustness and stability increase as the affinity exponent *a* increases. In this case, we know that homophyly/kinship and reciprocity are contradictory. To understand this, we recall Darwin’s proposal in his theory of evolution:

1. Individual fitness is key to survival.
2. Animals form social groups in which individuals cooperate among each other, referred to as Darwinian cooperation.
3. Kinship and reciprocity are the means of individual fitness.

Our results imply that Darwinian cooperation may only be achieved by a trade-off between homophyly/kinship and reciprocity and that a trade-off between homophyly/kinship and reciprocity is determined by the affinity exponent *a*. Therefore, the affinity exponent *a* opens the window for a balance between homophyly/kinship and reciprocity, and for achieving Darwin’s proposal above.

### Inclusiveness and external de-centrality of natural communities

Given two communities *X* and *Y*, let *e*(*X, Y*) be the number of edges between *X* and *Y*.

Let *G* be a network of our model and *X* be a natural community of *G*. We define the *inclusiveness of X* by


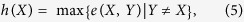


where *Y* ranges over all of the natural communities.

**Figure 6:**
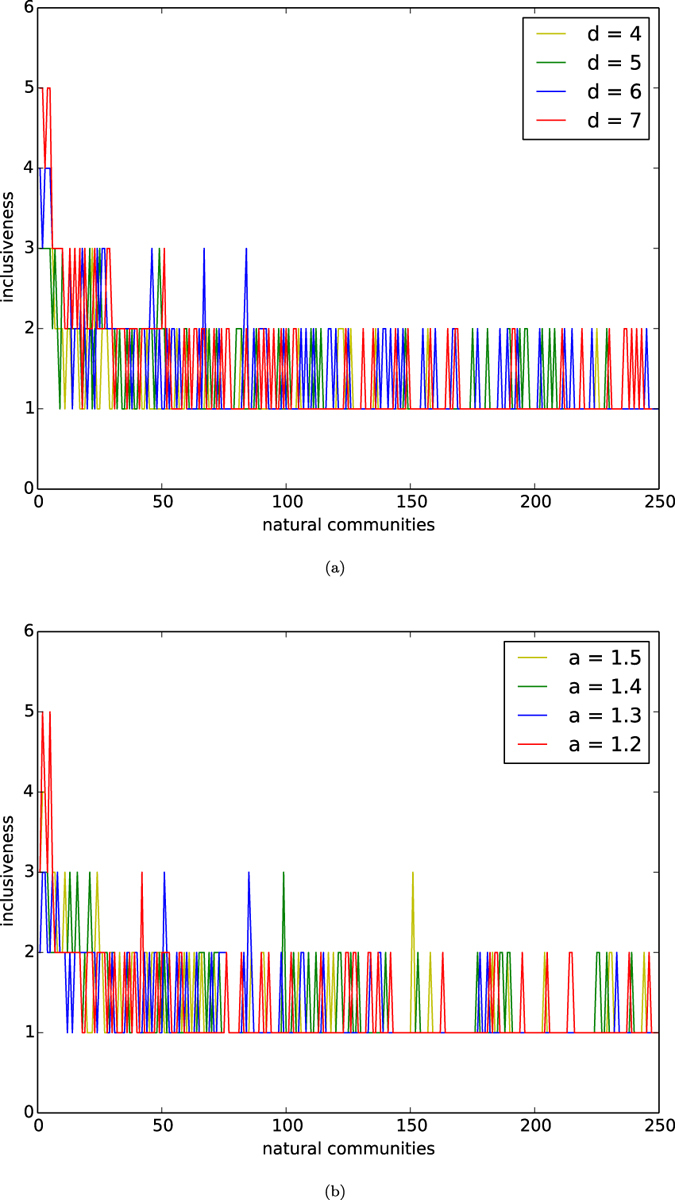
Inclusiveness. (**a**) Inclusiveness of natural communities of networks of the homophyly model. The curves are the distributions of reciprocity or networks of the model with *n* = 10,000, the affinity exponent *a* = 1.5, and *d* = 4, 5, 6, 7. (**b**) Inclusiveness of natural communities of networks of the homophyly model. The curves are the distributions of reciprocity or networks of the model with *n* = 10,000, *d* = 5, and the affinity exponents *a* = 1.2, 1.3, 1.4, 1.5.

In Fig. 6(a), we depict the distributions of inclusiveness of natural communities of networks of the homophyly/kinship model with *n* = 10,000, affinity exponent *a* = 1.5, and *d* = 4, 5, 6, 7. In Fig. 6(b), we depict the distributions of inclusiveness of natural communities of networks of our model with *n* = 10,000, *d* = 5, and affinity exponents *a* = 1.2, 1.3, 1.4, 1.5.

By observing Fig. 6(a,b), we demonstrate the following results:

1. For any *d* and affinity exponent *a*, almost all natural communities have inclusiveness 1,
2. There is a small number of natural communities having inclusiveness 2, and
3. There are a few communities having inclusiveness greater than 2.

The results imply that neighbours of a natural community *X* that are outside *X* are homogenously distributed among different natural communities. This finding can be explained as an interesting phenomenon of external de-centrality of natural communities. This property is the key to peaceful co-existence among the natural communities of a society.

### Widths of natural communities

Let *G* be a network and *X* be a natural community of *G*. We defined the *width of X in G*, denoted by width(*X*), to be the number of natural community *Y*’s such that *Y* ≠ *X* and there are edges between nodes in *X* and *Y*.

**Figure 7:**
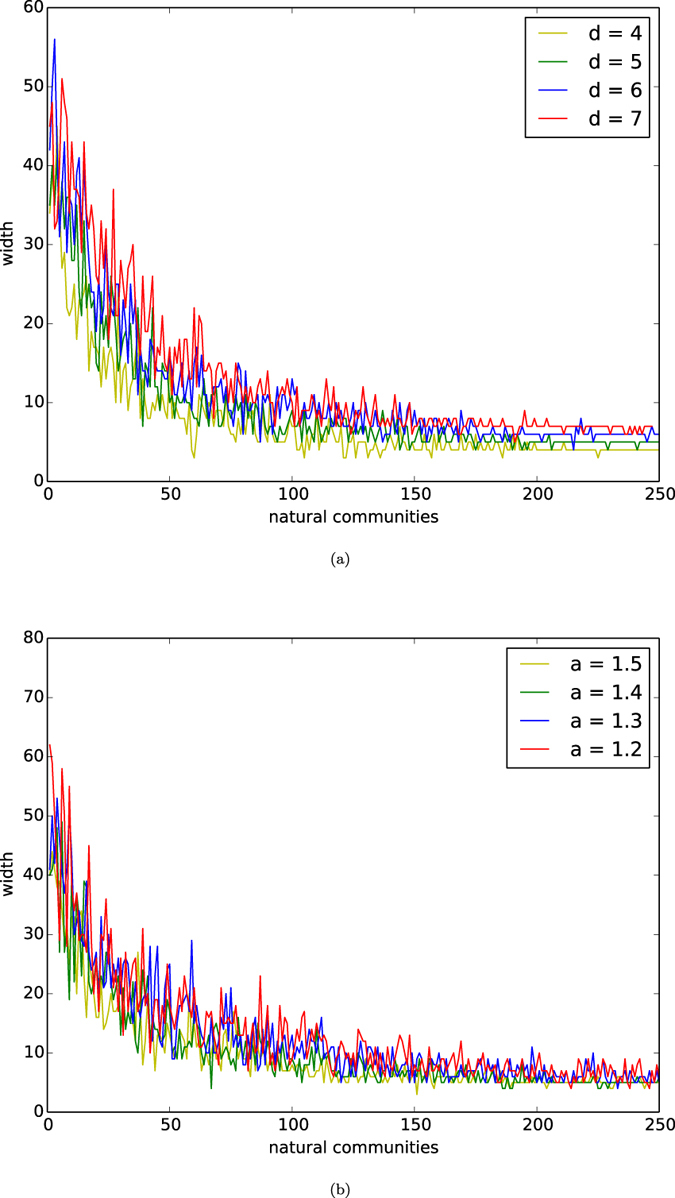
Widths. (**a**) Widths of natural communities of networks of the homophyly model. The curves are the distributions of reciprocity or networks of the model with *n* = 10,000, the affinity exponent *a* = 1.5, and *d* = 4, 5, 6, 7. (**b**) Widths of natural communities of networks of the homophyly model. The curves are the distributions of reciprocity or networks of the model with *n* = 10,000, *d* = 5, and the affinity exponents *a* = 1.2, 1.3, 1.4, 1.5.

In Fig. 7(a), we depict the distributions of widths of the natural communities of networks of the homophyly/kinship model with *n* = 10,000, affinity exponent *a* = 1.5, and *d* = 4, 5, 6, 7. In Fig. 7(b), we depict the distributions of widths of the natural communities of networks of our model with *n* = 10,000, *d* = 5, and affinity exponents *a* = 1.2, 1.3, 1.4, 1.5.

By observing Fig. 7(a,b), we demonstrate that the distributions of the widths of natural communities are similar to the distributions of reciprocities of the natural communities. This property is also the result of the inclusiveness properties of the networks.

Clearly, all the properties above provide insights for analysing natural communities.

## Realising Darwin’s Proposal by the Affinity Exponent

Recall Darwin’s proposal:

- Individual fitness is key to survival.
- Animals form social groups in which most individuals cooperate among each other.
- Kinship and reciprocity are the means of individual fitness.

The fundamental challenge is that the items above appear contradictory. Our analyses above suggest that these items could be consistent through an appropriate choice of affinity exponent *a*. To verify this hypothesis, we consider the evolutionary prisoner’s dilemma (PD, for short) games in the networks of our homophyly/kinship model.

In a PD game, the two players simultaneously decide their strategy, *C* (to cooperate) or *D* (to defect). For mutual cooperation, both players receive the payoff *R*, and they receive *P* upon mutual defection. If one cooperates and the other defects, the cooperator gains payoff *S*, and the traitor gains temptation *T*. The payoff rank for the PD game is given by *T* > *R* > *P* ≥ *S*.

Nowak and May^28^ proposed a simplified prisoner’s dilemma game by choosing *R* = 1, *P* = *S* = 0, and *T* = *b* for some *b* with 1 < *b* < 2.

We will investigate the emergence of cooperation in the weak prisoner’s dilemma games in the networks of our homophyly/kinship model. The experimental method is described in the supplementary information.

**Figure 8:**
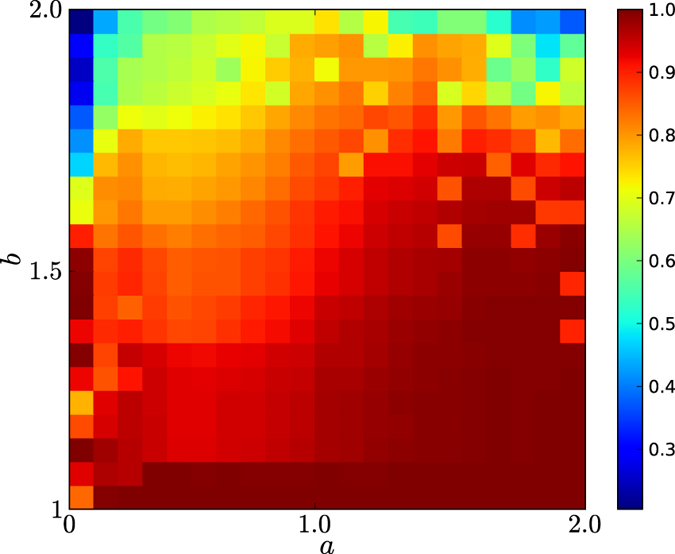
Emergence of cooperation of evolutionary prisoner’s dilemma games on networks of the homophyly model for *d* = 4. The number of nodes of the networks is 10,000. The equilibrium frequency is the average cooperation ratio of the last 1,000 steps of 3,000 steps for each of 50 evolutions for each of 50 networks of the same type. The color codes are from 0 (blue) to 1 (red). The updating strategy is the Fermi rule for randomly picked neighbors of nodes. The initial probability that a node takes strategy *C*, cooperator is *ε* = 0.3.

**Figure 9:**
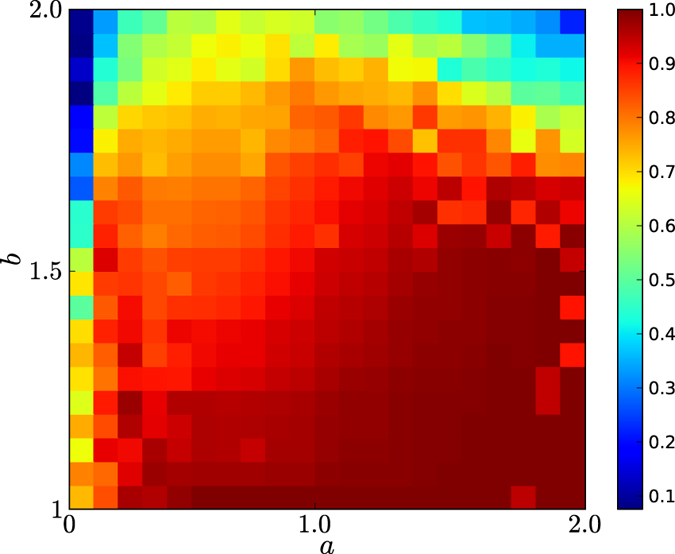
Emergence of cooperation of evolutionary prisoner’s dilemma games on networks of the homophyly model for *d* = 6. The experimental method is the same as Fig. 8.

In Figs 8 and 9, we depict the 2-dimensional colour codes of emergence of cooperation, i.e., *θ*(*C*) for networks of our natural selection model with *a* from 0 to 2 with unit 0.1, for all games with *b* from 1 to 2 with unit 0.1. In this experiment, for every type, we generated 50 networks, each of which implemented 50 evolutions of the games. The networks in Figs 8 and 9 have types *d* = 4 and 6, respectively. The colour codes are from 0 to 1, which is the equilibrium frequencies of cooperation in evolutionary prisoner’s dilemma games in the networks of our model.

By observing Figs 8 and 9, we demonstrate the following results:

1. For *d* = 4. According to Fig. 8, there is approximately a quadratic curve *f*(*a*), such that *f*(*a*) achieves a maximum value close to 2 at *a* = 1, such that for any *b*, if *b* ≤ *f*(*a*), cooperation quickly emerges in evolutionary prisoner’s dilemma games in networks of our model. If *b* is above the curve *f*(*a*), cooperation fails to emerge in evolutionary prisoner’s dilemma games in the networks.
2. For *d* = 4. According to Fig. 8, the first column contains the colour codes for the equilibrium frequencies of the networks of our model with *a* = 0, which are exactly the networks of the preferential attachment model. Based on the colour codes of the first column, we know that for *d* = 4, if *b* ≤ 1.5, cooperation is highly likely to emerge; however, if *b* > 1.5, cooperation is unlikely to emerge.
3. For *d* = 4, for any *a* and *b*, if *a* < 1 and *b* < *f*(*a*), the colour code of the equilibrium frequencies of cooperators is red, corresponding to a value of approximately 0.9, and if *a* ≥ 1 and *b* < *f*(*a*), the colour code of the cooperation is deep red, corresponding to value ≈1.
4. For *d* = 6. According to Fig. 9, there is approximately a quadratic curve *f*(*a*) such that *f*(*a*) achieves a maximum value close to 1.8 at *a* = 1, such that for any *b*, if *b* ≤ *f*(*a*), cooperation quickly emerges in evolutionary prisoner’s dilemma games in the networks of our model. If *b* is above the curve *f*(*a*), cooperation fails to emerge in evolutionary prisoner’s dilemma games on the networks.
5. For *d* = 6. According to Fig. 9, the first column contains the colour codes for the equilibrium frequencies of the networks of our model with *a* = 0, which are exactly the networks of the preferential attachment model. Based on the colour codes of the first column, we know that for *d* = 6, cooperation is unlikely to emerge in evolutionary prisoner’s dilemma games on the networks. This finding indicates that the emergence of cooperation in evolutionary games in the networks of the PA model is easily perturbed by the choices of *d*’s, and in particular, for large *d*’s, cooperation is unlikely to emerge.
6. For *d* = 6. According to Fig. 9, for any *a* and *b*, if 0.1 ≤ *a* < 1 and *b* < *f*(*a*), the equilibrium frequencies of cooperation are approximately 0.9, and if *a* ≥ 1 and *b* < *f*(*a*), the equilibrium frequencies of cooperation are ≈1.
7. According to Figs 8 and 9, for any *a* ≥ 0.1, the equilibrium frequencies of cooperations for any *b* are higher than those for *a* = 0. This finding indicates that the equilibrium frequencies of cooperation for evolutionary prisoner’s dilemma games in the networks of our homophyly/kinship model with *a* > 0 are always remarkably larger than those on the networks of the preferential attachment model (when *a* = 0 for our model). Consequently, we demonstrate that homophyly/kinship is essential to the emergence of cooperation in evolutionary prisoner’s dilemma games and that reciprocity alone in the heterogeneous networks of the PA model plays only a very limited role in the emergence of cooperation in evolutionary prisoner’s dilemma games.
8. According to Figs 8 and 9, if *a* is close to 2, there is a large *b*_0_, such that if *b* ≥ *b*_0_, cooperation is unlikely to quickly emerge in the evolutionary games. This finding indicates that homophyly/kinship alone fails to guarantee cooperation.
9. The colour codes for the block *a* < 1 and the block *a* ≥ 1 are distinguishable in the sense that the former is slightly weaker than the latter. This finding indicates that *a* = 1 could be a threshold for the evolution of networks, with interesting implications.
10. In both cases, if *a* is either too small or too large, there is a *b*_0_, such that *b*_0_ is not large, and for any *b*, if *b* ≥ *b*_0_, cooperation fails to quickly emerge in evolutionary prisoner’s dilemma games in the networks of our model. If *a* = 1, cooperation quickly emerges in evolutionary prisoner’s dilemma games in the networks of our model, unless *b* is too large.

Our results demonstrate that both homophyly/kinship and reciprocity are essential to the emergence of cooperation, that for a nontrivial choice of affinity exponent *a*, there is a large *b*_0_, such that if *b* < *b*_0_, cooperation emerges, and that for *a* ≈ 1, cooperation emerges for almost all *b*’s. The results validate that Darwin’s proposal is perfectly correct, which can be realised by an appropriately chosen affinity exponent *a*. This finding is a surprising discovery in the application of our model to understanding the emergence of cooperation in evolutionary games in networks. We remark that there must exist more notable applications of the model.

Finally, we remark that our results prompt more questions than answers. The first question is to mathematically prove the experimental discovery above; the second is to understand the reason why *a* = 1 plays the role of a threshold for the emergence of cooperation in evolutionary prisoner’s dilemma games in the networks; and the third is to fully develop evolutionary game theory. Our model provides the first significant step towards answering these questions.

## Structure Entropy Minimisation Principle for Detecting Natural Communities

The first principle explored by our homophyly/kinship model is that nodes of a network form natural communities for which homophyly/kinship is the mechanism. Consequently, individuals of a natural community share common attributes and form a functional module of the network. This principle is completely different from the existing approaches to algorithmic communities, which simply interpret the outputs of reasonable algorithms as communities.

Therefore, the immediate question is the following: Can we find the natural communities in a real world network? Before answering this question, we examined two well-known algorithms. The first is the algorithm frequently used by Clauset, Newman and Moore^29^, denoted by 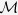, based on modularity. The second is the algorithm by Rosvall and Bergstrom^30^, denoted by InforMap, which was developed based on information compression. Clearly, the two algorithms are completely different.

We checked whether the algorithms could find the natural communities of the networks generated by our homophyly/kinship model.

To measure the precision of a community finding algorithm, we introduced some notations.

Given a network *G* = (*V, E*), let *X* and *Y* be two subsets of *V*. We defined the *similarity of X and Y* by


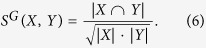


Suppose that 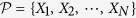 and 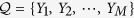 are two partitions of *G*.

Then, we defined the *similarity of* 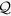 *to* 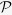 to be the function 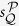 such that for all 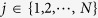,


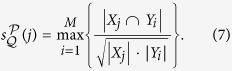


In Table 1, we describe the similarities of the natural communities of networks of our model found by the algorithm 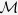 based on modularity.

In Table 2, we describe the distributions of similarities of the communities of the networks of our model found by the algorithm InforMap.

By observing Tables 1 and 2, we demonstrate that neither the algorithm 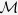 nor the InforMap identified the natural communities of the networks of our model, especially for *a* ≤ 1. Therefore, the existing algorithms, although well-known, were unable to identify the natural communities.

Li, Li and Pan^25^ proposed such a new algorithm for detecting natural communities. The algorithm is based on our new notion of structure entropy of graphs and is an information theoretical measure of the quality of community structures of networks, which we introduce below.

Given a graph *G* = (*V, E*), suppose that 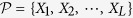 is a partition of *V*. We define the *structure entropy of G by* 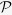 as follows:


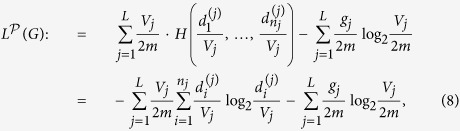


where *L* is the number of modules in partition 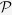, *n*_*j*_ is the number of nodes in module *X*_*j*_, 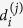 is the degree of the *i*-th node of *X*_*j*_, *V*_*j*_ is the volume of module *X*_*j*_, and *g*_*j*_ is the number of edges with exactly one endpoint in module *X*_*j*_.

The intuition of 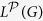 is as follows. Given the partition 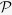 of *G*, a node *v* of *G*, say, is encoded by a pair of codes (*j, i*), such that *j* is the code of the community *X* containing *v* and is called global code, and *i* is the code of *v* in its community *X*, and is called the local code. In the definition of 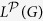, the first term is the least number of bits to determine the local code of the node that is accessible from a step of random walk in *G* with stationary distribution, and the second term is the least number of bits to determine the global code of the node that is accessible from a step of random walk from a node outside the community. Therefore, 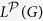 is the number of bits needed to determine the code (*j, i*) of the node *v* that is accessible from a step of random walk in *G* by using the partition 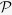.

Next, we defined the *structure entropy*, also referred to as *module entropy* or *local positioning entropy*, of *G* as follows:


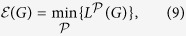


where 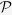 runs over all the partitions of *G*.

Given a network *G* = (*V, E*), it is a a challenging problem to find a partition 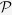, such that 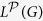 is minimised.

Here, we describe a simple greedy algorithm to find a partition that minimises the structure entropy of the network *G*. Before describing the algorithm, we defined the following notation.

Suppose that 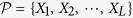 is a partition of *V*. For *i, j* with 1 ≤ *i, j* ≤ *L*, we define 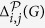 as follows:


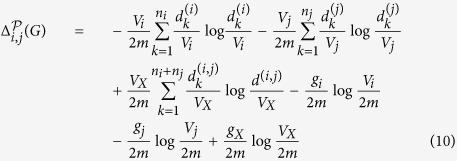



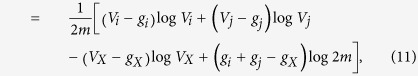


where *X* = *X*_*i*_ 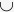 *X*_*j*_, *V*_*X*_ is the volume of *X, g*_*X*_ is the number of edges from *X* to nodes outside *X*, and 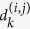 is the degree of the *k*-th node in *X*.

By definition, 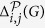 is locally computable.

If there is no edge between *X*_*i*_ and *X*_*j*_, then *g*_*X*_ = *g*_*i*_ + *g*_*j*_. In this case,


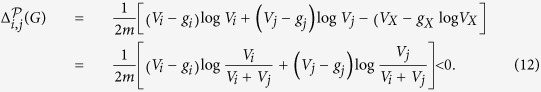


Our algorithm, denoted by 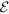, proceeds as follows.

1. Suppose that 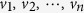 are all the nodes in *V* with ordering as they are listed. Set *X*_*i*_ to be the singleton {*v*_*i*_} for all *i*, which form the initial partition of *V*. Suppose that 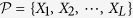 is a partition with ordering as they are listed.
2. If there is no *i* < *j*, such that 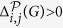, terminate with output 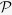.
3. Otherwise, let *i*_0_, *j*_0_ be such that 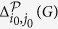 is maximised among 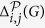 for all *i, j*’s, set 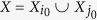, set 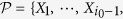 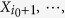 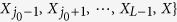, and go back to step (2).

In Table 3, we describe the distributions of similarities of the natural communities of networks of our model found by our algorithm 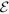.

By observing Table 3 and by comparing Tables 3, 2 and 1, we demonstrate the following findings:

1. if *a* ≤ 1, our algorithm 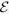 is remarkably better than the InforMap,
2. if *a* > 1, 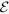 and InforMap are equally successful in identifying the natural communities.
3. for any *a*, both 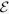 and InforMap are remarkably better than 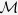.

Therefore, our algorithm 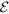 always defects 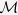, and in certain cases defects InforMap in identifying natural communities. All the existing algorithms are similar to 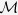 and InforMap, with similar or slightly better performance (for the most part in maximising modularity). We thus believe our algorithm 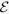 is currently the best algorithm for finding natural or true communities. In addition, our algorithm 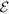 is based on our new theory of the structure entropy of graphs, providing a large room for further improvement of the algorithm on the basis of minimisation of the structure entropy of networks.

We then applied algorithm 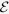 to detect natural communities in real world networks.

## Detecting Cell Types of Normal Tissues

We have observed that our algorithm 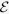 identifies or precisely approximates almost all natural communities of the networks of our homophyly/kinship model.

Can algorithm 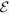 identify true functional modules in the real world? The answer is affirmative. We verified our result by developing a gene map of cell types and by defining the cell modules by gene expression patterns for normal tissues.

First, we constructed a cell sample network on the basis of gene expression profiles. We used the dataset for normal tissues described in Su *et al.*^31^, which uses a simple signal-to-noise ratio (SNR) to rank genes. The final gene pool was obtained by selecting the most up-regulated genes for each class, where the exact number depended on the original dataset described in Ramaswamy *et al.*^32^. The data contain the expression of 1,277 genes for 90 samples, which form 13 cell types^31^. The 13 distinct tissue types are: breast (5), prostate (9), lung (7), colon (11), germinal centre cells (6), bladder (7), uterus (6), peripheral blood monocytes (5), kidney (12), pancreas (10), ovary (4), whole brain (5), and cerebellum (3), where the number following the type is the number of cells in the type. We chose *k* = 3 for the construction of the gene network for the normal tissues.

Our gene map of a classification of the cell sample network was defined using all the genes, which gives rise to a global picture of the gene expression profiles of the classification. Details are provided in the supplementary information.

**Figure 10:**
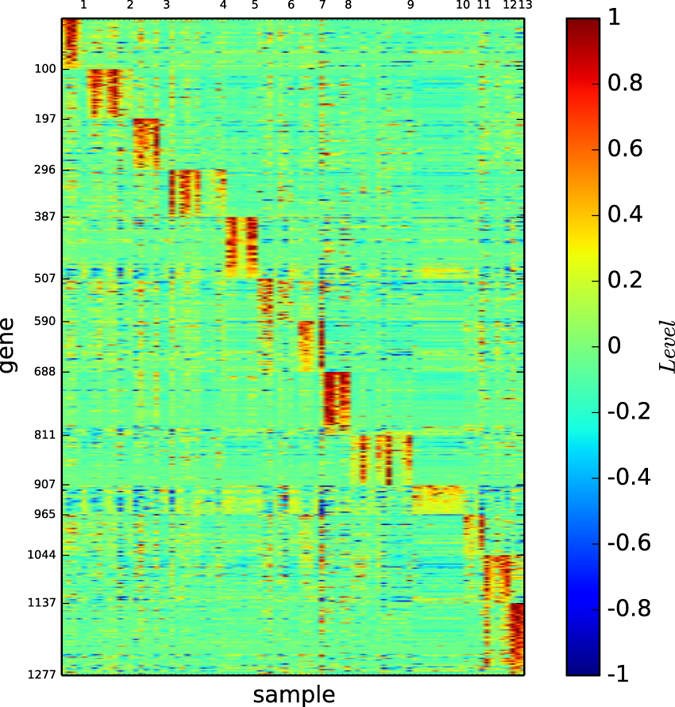


In Fig. 10, we depict the gene map of the 13 true cell types of normal tissues with ordering as listed by: breast, prostate, lung, colon, germinal, bladder, uterus, peripheral, kidney, pancreas, ovary, whole and cerebellum.

By observing Fig. 10, we demonstrate the following results:

1. All the types are distinguishable by the gene map.
2. Bladder, pancreas and ovary are not remarkably expressed, but all the others are remarkably expressed by the corresponding gene expression patterns.

The similarities of the true cell types of the normal tissues found by our algorithm 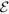 are given in the supplementary information. This finding shows that our algorithm 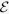 exactly identifies or precisely approximates the true cell types of the normal tissues; details are provided in the supplementary information.

**Figure 11:**
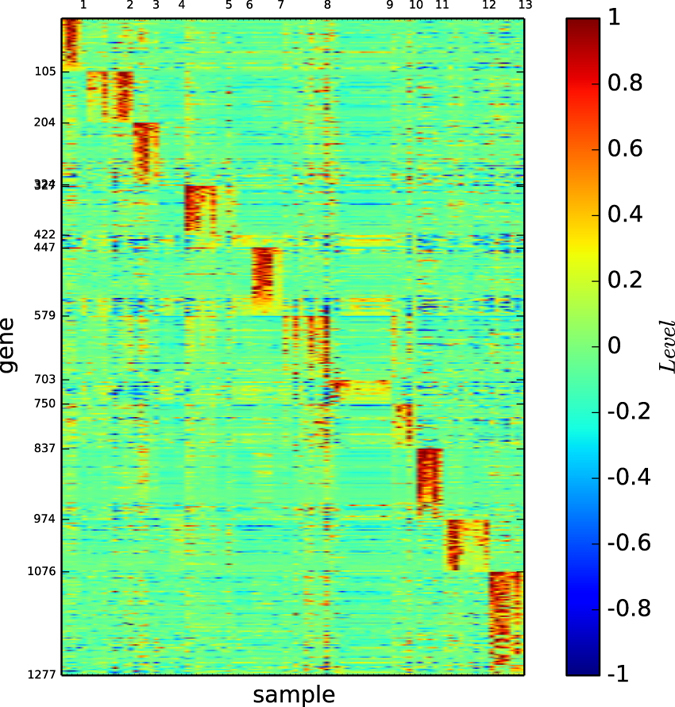
Gene map of the classification of the gene expression network of the normal tissues, found by our algorithm 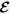
.

In Fig. 11, we depict the gene map of the classification of normal tissues given by algorithm 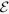.

By observing Fig. 11, we demonstrate the following results:

1. Communities 1, 2, 7 and 11 are exactly the breast, prostate, germinal and peripheral, respectively.
2. Communities 3, 5, 9, 10 and 12 are basically the types lung, colon, pancreas, ovary, and kidney, respectively. Each of these communities is remarkably expressed by a gene expression pattern.
3. Community 4 consists of a few lung and kidney cells. This community is not well-expressed by the gene map.
4. Community 6 is a combination of some colon and uterus cells, which is not well-expressed by the gene map.
5. Communities 8 is a combination of bladder, uterus, and whole, which is remarkably expressed by the gene map.
6. Community 13 is a combination of whole and cerebellum, which is remarkably expressed by the gene map.
7. Our algorithm exactly identifies or approximates the true cell types well. We say that a community *X* is well-defined if there is a gene set *B*, such that genes in *B* remarkably express *X*, but fail to express any community *Y* other than *X*.
8. Except for communities 4 and 6, all the communities found by our algorithm are well-defined. This gives rise to a high-definition and one-to-one map between the found communities and the gene expression patterns, meaning that a gene set determines a community, or a community is uniquely determined by a gene set, i.e., a gene expression pattern.

The results above show that the modules of the cell sample network of the normal tissues found by our algorithm are uniquely determined by gene expression patterns. That is, every module has a unique gene set that remarkably expresses the module and fails to express any other modules. Therefore, well-defined communities are indeed functional modules, playing a role and sharing common attributes. We noted that the gene map of the classification found by our algorithm was even better than the true cell types, in the sense that the high gene expression profiles were more concentrated in the diagonal blocks of gene expression patterns and communities.

We therefore conclude that natural communities of real world networks, if detected or well-approximated, are indeed functional modules by roles, and that our algorithm 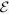 does accurately identify or precisely approximate true functional modules of some real world networks.

[**Remark**: Li, Yin and Pan have shown that the algorithm 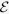 identifies cell types and subtypes for five cancers, such that the types and subtypes identified by the algorithm are definable by a unique gene expression pattern. Furthermore, by using clinical data, it has been shown that most cell samples within the same type or subtype identified by 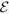 share similar survival times, survival indicators, and International Prognostic Index (IPI) scores, and that cell samples of different types and subtypes identified by the algorithm 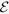 have distinct overall survival times, survival ratios and IPI scores. In addition, it was shown that our algorithms on the basis of structure entropy minimisation are the only ones that passed the test of clinical interpretability and distinction for classification of cancers. This achievement will be published separately.]

## Characteristic Properties of Natural Communities of Real World Networks

We have seen that the natural communities of networks of our homophyly/kinship model satisfy a number of characteristic properties, including robustness, stability and reciprocity. Are these properties shared by real world networks? We answered this question affirmatively by analysing the communities found by our algorithm 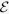 for a citation network and a protein interaction network.

### Citation Network HEPPH

This is the Arxiv HEP-PH (high energy physics applications), containing 34,401 papers, and 421,485 citations.

**Figure 12:**
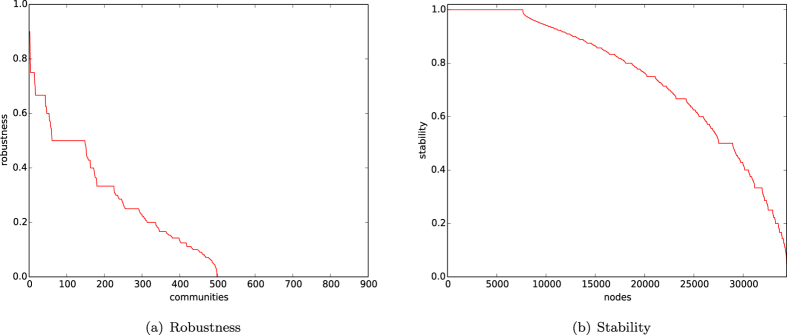
Citation graph HEP-PH. (**a**) Robustness of the communities found by our algorithm. (**b**) Stability of the networks given by the communities found by our algorithm.

In Fig. 12(a,b), we depict the robustness and stability of the communities of the citation network HEPPH found by our algorithm 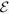, respectively. The curves in Fig. 12 are defined decreasingly.

1. From Fig. 12(a), we demonstrate that there are approximately 50 communities in which 70% papers have citation links to papers only within their own communities, that there are more than 150 communities in which at least 50% papers have citation links to papers only within their own communities, and that there are more than 250 communities in which at least 30% papers have citation links only within their own communities.
2. From Fig. 12(b), we know that there are 25% of papers having stability ≈1, and that there are more than 60% of papers having stability greater than or equal to 0.6.

(1) indicates that the citation network HEPPH does have some robustness. However, the robustness of the found communities is not very high. The reasons could be that for academic research, a paper usually cites all the related references, and some of them could belong to distinct topics. This is the nature of citation networks. (2) shows that the majority of references for most papers belong to their own topics. Nevertheless, (1) and (2) show the robustness and stability of the citation network.

**Figure 13:**
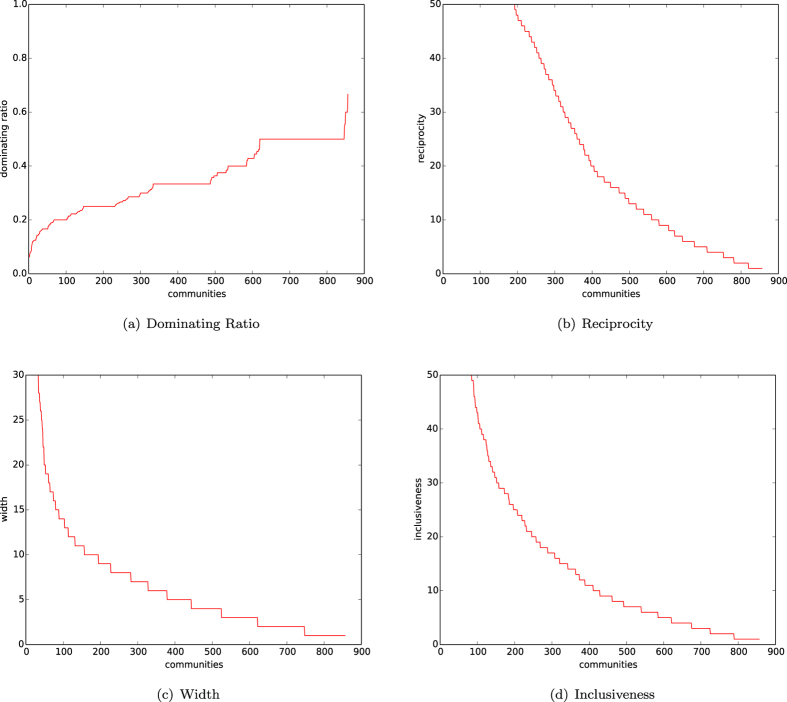
Citation graph HEP-PH. (**a**) Dominating ratio. (**b**) Reciprocity. (**c**) Widths. (**d**) Inclusiveness.

In Fig. 13(a–d), we depict the distributions of the dominating ratio, reciprocity, width and inclusiveness of the communities of the citation network found by algorithm 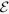. This figure demonstrates the following results.

1. The curve of the dominating ratio is less than 0.2 for half of the communities, and less than 0.3 for most communities.
2. The curves of the reciprocity, width, and inclusiveness are all similar.
3. The curve of reciprocity of the citation network is significantly high.
4. The curve of reciprocity of the citation network is similar to that of the networks of our homophyly model with affinity exponent *a* ≥ 1.

By (1), we know that most communities have a dominating set of size 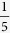 of that of the communities, meaning that most communities are heterogeneous, and that a recommendation of a dominating set of sizes 20% of that of the communities would be sufficient for a search engine. (2)–(4) show that the citation network has high reciprocity, and that the curve of the reciprocity actually follows the law of networks of our homophyly/kinship model.

### Yeast network

The protein interaction network data (for yeast) is from Jeong *et al.*^33^; the largest connected component of the network contains 1,458 nodes and 1,948 interactions.

**Figure 14:**
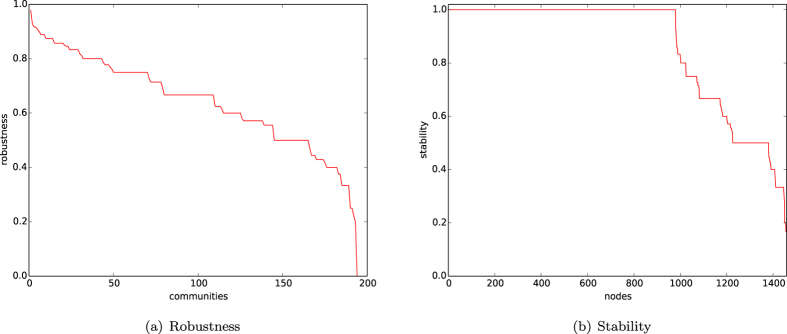
Yeast network. (**a**) Robustness of the communities found by our algorithm. (**b**) Stability of the networks given by the communities found by our algorithm.

In Fig. 14(a,b), we depict the robustness and stability of the communities of the metabolic network found by our algorithm 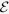, respectively.

Figure 13 demonstrates the following:

1. There are 50 communities with robustness greater than or equal to 0.8, 100 communities with robustness greater than 0.7, and 150 communities with robustness greater than or equal to 0.5.
2. There are 1,000 nodes with stabilities ≈1, and 1,300 nodes with stabilities larger than or equal to 0.5.

(1) and (2) show that the protein network has high robustness and stability.

**Figure 15:**
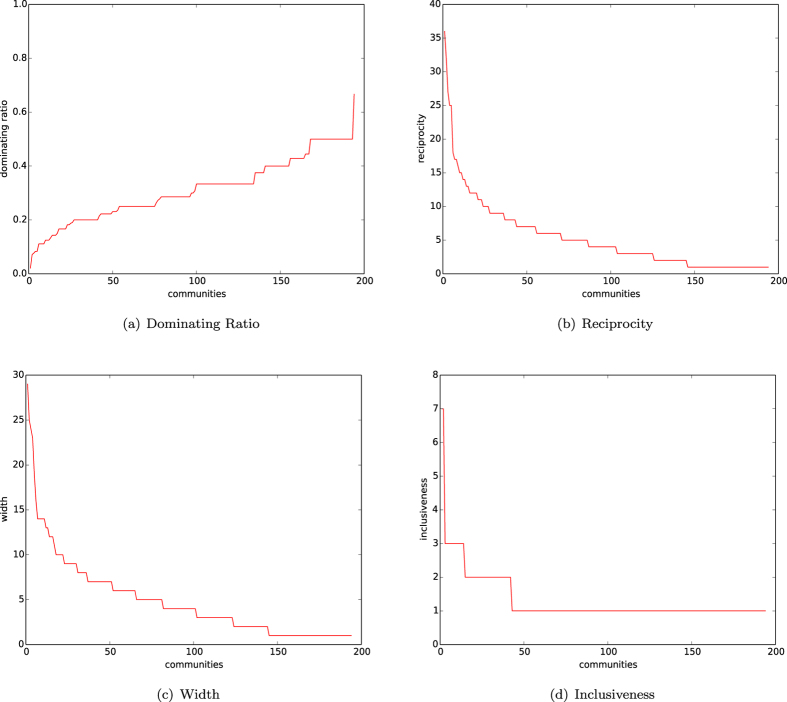
Yeast network. (**a**) Dominating ratio. (**b**) Reciprocity. (**c**) Widths. (**d**) Inclusiveness.

In Fig. 15(a–d), we depict the distributions of the dominating ratio, reciprocity, width and inclusiveness of the communities of the yeast network found by algorithm 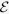. The figure demonstrates the following results.

1. There are 50 communities with a dominating ratio less than 0.2, 100 communities with a dominating ratio less than or equal to 0.3 and 150 communities with a dominating ratio less than or equal to 0.4.
2. The curves of the reciprocity, width, and inclusiveness are all similar.
3. The curve of reciprocity of the protein network is high.
4. The curve of reciprocity of the protein network is similar to that of the networks of our homophyly/kinship model with affinity exponent *a* > 1.

As before, by (1), we know that the found communities of the protein network are heterogeneous, each of which may be well-represented by a small dominating set of the communities. (2)–(4) demonstrate that the protein network does have a significant reciprocity, following the law of the networks of the homophyly/kinship model.

We thus verified that the characteristic properties of the networks of our homophyly/kinship model are all shared by real world networks, including both social networks and biological networks.

## Methods

The data of the citation network HEP-PH in our experiments can be found at the websites: http://snap.standford.edu, and http://www-personal.umich.edu/~mejn/netdata. The protein interaction network data (for yeast) can be found at the website http://www3.nd.edu/~networks/resources.htm.

## Conclusions and Discussions

We proposed a hypothesis that homophyly/kinship and the roles and survival of individuals are the intrinsic mechanisms of the formation of natural communities in nature and society. Based on this hypothesis, we proposed a homophyly/kinship model of networks by introducing the notion of the affinity exponent. We demonstrated that networks generated by our homophyly/kinship model satisfy a number of characteristic properties, among which robustness, stability and reciprocity are the most interesting new properties. We showed that robustness/stability increase as the affinity exponent *a* increases, and that reciprocity decreases as the affinity exponent *a* increases. By using this result, we analysed and verified that the affinity exponent allows for the realisation of Darwin’s proposal that homophyly/kinship and reciprocity are the strategies of individual fitness that lead to Darwinian cooperation, i.e., most individuals of a true social group cooperate with each other. We proposed a new algorithm based on our new notion of the structure entropy of graphs. We demonstrated that our algorithm exactly identifies or precisely approximates natural or true communities in both networks of our homophyly/kinship model and real world networks, such as the gene expression network of normal tissues. We verified that communities found by our algorithm share all the characteristic properties of networks explored from our homophyly/kinship model, by using some previous experiments for metabolic networks, a citation network and a protein interaction network. Our experiments on real world networks show that many natural communities of real world networks have a dominating set of 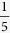 of the sizes of the communities. This property may have interesting applications in the recommendation of systems.

## Additional Information

**How to cite this article**: Li, A. *et al.* Homophyly/Kinship Model: Naturally Evolving Networks. *Sci. Rep.* **5**, 15140; doi: 10.1038/srep15140 (2015).

## Supplementary Material

Supplementary Information

## Figures and Tables

**Table 1:**
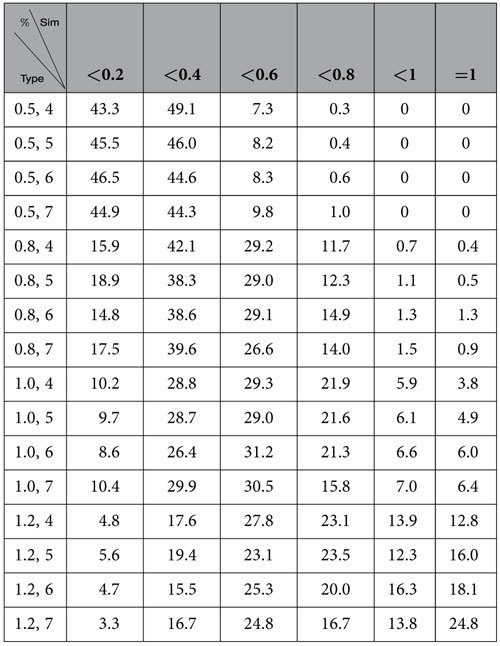
Similarity of communities found by 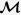 for networks of the homophyly/kinship model with *n* = 10,000, *d* = 4, 5, 6, 7 and *a* = 0.5, 0.8, 1, 1.2.

**Table 2:**
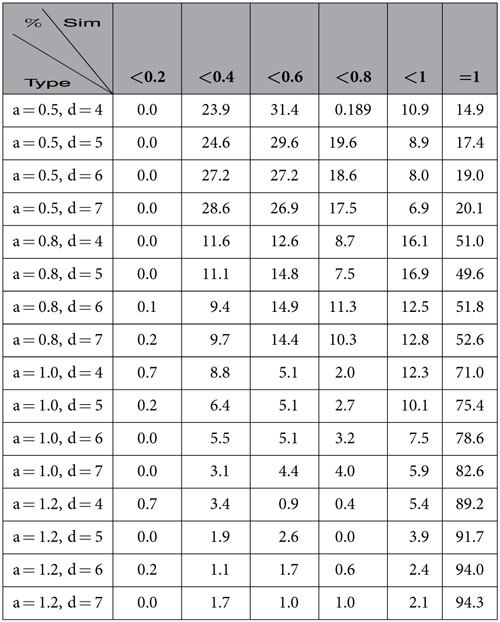
Similarity of the communities found by the algorithm Informap for networks of the homophyly/kinship model with *n* = 10,000, *d* = 4, 5, 6, 7 and *a* = 0.5, 0.8, 1, 1.2.

**Table 3:**
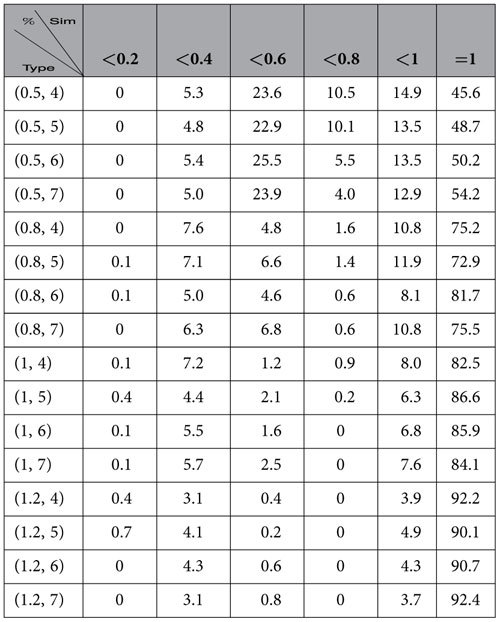
Similarity of communities found by 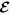 for networks of the homophyly/kinship model, with *d* = 4, 5, 6, 7, *a* = 0.5, 0.8, 1, 1.2 and *n* = 10,000.
