# Supplementary material for: Homophyly/Kinship Model: Naturally Evolving Networks
Source: Sci Rep. 2015 Oct 19;5:15140. doi: 10.1038/srep15140 (PMC4609949; doi:10.1038/srep15140)
Supplement: Supplementary Information [file srep15140-s1.pdf]

Homophyly/Kinship Model: Naturally Evolving Networks (Supplementary Information)

Angsheng Li[1,⋆], Jiankou Li[1,2], Yicheng Pan[1,3], Xianchen Yin[1,2], and Xi Yong[1,2]

[1] *State Key Laboratory of Computer Science, Institute of Software, Chinese Academy of Sciences, Beijing, 100190, P. R. China*

[2] *University of Chinese Academy of Sciences, Beijing, P. R. China*

[3] *State Key Laboratory of Information Security, Institute of Information Engineering, Chinese Academy of Sciences, Beijing, P. R. China*

[⋆] *Correspondence: angsheng@ios.ac.cn*

We establish the mathematical principles of the networks of the homophyly/kinship model, consisting of a self-organising law, a holographic law, a power law, a small community phenomenon, a local communication law, a small diameter property, a natural community law, a degree priority law, a king node law, an inclusion-exclusion law, and a reciprocity law etc of the networks of the homophyly/kinship model etc. We introduce the evolutionary games implemented in our paper, and describe the method of gene map of cell modules for our experiment on the gene expression network of normal tissues, and the classification of cell types of the normal tissues found by our algorithm.

Before proving the results, we introduce some useful probabilistic tools and inequalities, which will be frequently used throughout the proofs.

We will use the following form of Chernoff bound.

Lemma 1 *(Chernoff bound[1]) Let $X_1, \ldots, X_n$ be independent random variables with $\Pr[X_i = 1] = p_i$ and $\Pr[X_i = 0] = 1 - p_i$. Denote the sum by $X = \sum_{i=1}^{n} X_i$ with expectation $E(X) = \sum_{i=1}^{n} p_i$. Then we have*

$$\Pr[X \leq E(X) - \lambda] \leq \exp\left(-\frac{\lambda^2}{2E(X)}\right),$$

$$\Pr[X \geq E(X) + \lambda] \leq \exp\left(-\frac{\lambda^2}{2(E(X) + \lambda/3)}\right).$$

We will use the following form of Azuma's inequality for martingales.

Lemma 2 *(Azuma's inequality) Let $\mathbf{c} = (c_1, \ldots, c_n)$ be a vector of positive entries. Let a sequence of random variables $X_0, X_1, \ldots, X_n$ be a martingale. If it is **c**-Lipschitz, that is, $|X_i - X_{i-1}| \leq c_i$ for $i = 1, \ldots, n$, then for any $\lambda > 0$,*

$$\Pr[X_n \leq X_0 - \lambda] \leq \exp\left(-\frac{\lambda^2}{2\sum_{i=1}^{n} c_i^2}\right),$$

$$\Pr[X_n \geq X_0 + \lambda] \leq \exp\left(-\frac{\lambda^2}{2\sum_{i=1}^{n} c_i^2}\right).$$

We will use the following form of supermartingale inequality.

Lemma 3 *(Supermartingale inequality, Theorem 2.40[2]) For a filter $\{0, \Omega\} = \mathcal{F}_0 \subset \mathcal{F}_1 \subset \cdots \subset \mathcal{F}_n = \mathcal{F}$, suppose that a non-negative random variable X_i is $\mathcal{F}_i$-measurable for $0 \leq i \leq n$. Let B*

be the bad set associated with the following admissible conditions: (that is, the set of events that the conditions fail to hold.)

$$\begin{aligned} E(X_i|\mathcal{F}_{i-1}) &\leq X_{i-1}, \\ \mathit{Var}(X_i|\mathcal{F}_{i-1}) &\leq \sigma_i^2 + \phi_i X_{i-1}, \\ X_i - E(X_i|\mathcal{F}_{i-1}) &\leq a_i + M, \end{aligned}$$

where σ_i, ϕ_i, a_i and M are non-negative constants. Then we have

$$\Pr(X_n \geq X_0 + \lambda) \leq \exp\left(-\frac{\lambda^2}{2(\sum_{i=1}^n(\sigma_i^2 + a_i^2) + (X_0 + \lambda)(\sum_{i=1}^n \phi_i) + M\lambda/3)}\right) + \Pr(B).$$

The following fact will also be very useful in our proofs.

Fact 1 *For any real x,*

$$\frac{1}{x+1} \leq \log\left(1 + \frac{1}{x}\right) \leq \frac{1}{x}.$$

Note that $1 + y \leq e^y$ holds for all real y. The fact is obtained by replacing y with $-\frac{1}{x+1}$ and $\frac{1}{x}$, respectively.

The following expansion of power series is folklore.

Fact 2 *For any $u > 0$ and $|x| \leq 1$,*

$$\begin{aligned} (1 \pm x)^u &= 1 \pm ux + \frac{u(u-1)}{2!}x^2 \pm \frac{u(u-1)(u-2)}{3!}x^3 \\ &\quad + \cdots + (-1)^m \frac{u(u-1)\cdots(u-m+1)}{m!}x^m + \cdots . \end{aligned}$$

Fundamental Properties

We use $\mathcal{H}(n,a,d)$ to denote the set of networks of n nodes, constructed from the homophyly/kinship model with affinity exponent a and average number of edges d. Let $G=(V,E)\in\mathcal{H}(n,a,d)$ be a network of n nodes generated from our homophyly/kinship model. We use G_t to denote the graph obtained at the end of time step t of the construction of G, and C_t to denote the set of seed nodes of G_t. Recall that every node $v\in V$ is associated with a color. The vertices in V is partitioned naturally as the homochromatic sets, each of which is a connected set of nodes of the same color. Every homochromatic set contains a seed node, which is the first node of the community. For an edge $e=(u,v)$, we call e a *local edge*, if the two endpoints u,v share the same color, and a *global edge*, otherwise.

In this section, we will prove some basic properties about the number of seed nodes, the sizes, volumes and the numbers of global edges of homochromatic sets.

Theorem 1 *(Basic properties) Given $a\geq 0$, and $d\geq 2$, let $G=(V,E)$ be a graph of n nodes generated from our homophyly/kinship model. Let $T_1=\log^{a+1} n$ and $T_2=\frac{n}{\log^b n}$ for some positive constant b. Then the following properties hold:*

(1) With probability $1-o(1)$, for all $t\geq T_1$, $\frac{t}{2\log^a t}\leq |C_t|\leq \frac{2t}{\log^a t}$.

(2) For each homochromatic set S, if $t>t_S\geq T_1$, then the expectation of its size at time step t is $\Theta(\log^{a+1} t-\log^{a+1} t_S)$, where t_S is the time step at which the seed node of S is created.

(3) With probability $1 - o(1)$, every homochromatic set in G has a size upper bounded by $O(\log^{a+1} n)$.

(4) For each homochromatic set S, if $t_S \geq T_2$, then the number of global edges in G connected nodes in S, denoted by g_S, satisfies that, for sufficiently large n,

(i) if $a > 1$, then $E(g_S) \leq \frac{5}{2}(a+1)d^2b^2(\log\log n)^2$;

(ii) if $a = 1$, then $E(g_S) \leq 8d^2b^2(\log\log n)^2$; and

(iii) if $0 < a < 1$, then $E(g_S) \leq 5d^2b^2(\log\log n)^2$.

For (1). By the construction of G, the expectation of $|C_t|$ is

$$E[|C_t|] = 2 + \sum_{i=3}^{t} \frac{1}{\log^a i}.$$

By indefinite integral

$$\int (\frac{1}{\log^a x} - \frac{a}{\log^{a+1} x})dx = \frac{x}{\log^a x} + C,$$

we know that if $t \geq T_1$ is large enough, then

$$\begin{aligned}
\sum_{i=3}^{t} \frac{1}{\log^a i} &\leq 1 + \int_2^t \frac{1}{\log^a x}dx \\
&\leq \int_2^t \frac{6}{5}(\frac{1}{\log^a x} - \frac{a}{\log^{a+1} x})dx \\
&\leq \frac{4t}{3\log^a t},
\end{aligned}$$

where $\frac{6}{5}$ and $\frac{4}{3}$ are chosen arbitrarily among the numbers larger than 1. Similarly,

$$\begin{aligned}\sum_{i=3}^{t} \frac{1}{\log^a i} &\geq \int_2^t \frac{1}{\log^a x} dx \\ &\geq \int_2^t (\frac{1}{\log^a x} - \frac{a}{\log^{a+1} x}) dx \\ &\geq \frac{3t}{4 \log^a t}.\end{aligned}$$

By the Chernoff bound, since $t \geq T_1$, with probability $1 - exp(-\Omega(\frac{t}{\log^a t})) = 1 - o(n^{-1})$, we have $\frac{t}{2\log^a t} \leq |C_t| \leq \frac{2t}{\log^a t}$. By the union bound, such an inequality holds for all $t \geq T_1$ with probability $1 - o(1)$.

For (2). By the construction of G, the expectation of $|S|$ at time step t is

$$E(|S|) = 1 + \sum_{i=t_S+1}^{t} \left(1 - \frac{1}{\log^a i}\right) \cdot \frac{1}{|C_i|}.$$

Before going on the proofs, we define the following:

Definition 1 *Let $\mathcal{E}$ be the event that: for all $i \geq T_1$, $\frac{i}{2\log^a i} \leq |C_i| \leq \frac{2i}{\log^a i}$.*

We will use this event several times throughout the proofs.

By (1), we know that $\mathcal{E}$ holds with probability $1 - o(1)$. Thus, at time step t,

$$\begin{aligned} E(|S|) &= \Theta\left(\sum_{i=t_S}^{t}\left(1 - \frac{1}{\log^a t}\right) \cdot \frac{\log^a t}{t}\right) \\ &= \Theta\left(\int_{t_S}^{t} \frac{\log^a x}{x} dx\right) \\ &= \Theta(\log^{a+1} t - \log^{a+1} t_S). \end{aligned}$$

For (3). It suffices to prove that with probability $1 - o(n^{-1})$, the homochromatic set of the first color κ has size $O(\log^{a+1} n)$. Then the result follows from the union bound.

Let S_κ be the set of nodes share color κ.

Conditioned on the event $\mathcal{E}$, in Definition 1, for large enough n,

$$\begin{aligned} E(|S_\kappa|) &= 1 + \sum_{i=3}^{n}\left(1 - \frac{1}{\log^a i}\right) \cdot \frac{1}{|C_i|} \\ &\leq T_1 + \sum_{i=T_1+1}^{n}\left(1 - \frac{1}{\log^a i}\right) \cdot \frac{2\log^a i}{i} \\ &\leq 3\log^{a+1} n. \end{aligned}$$

By the Chernoff bound,

$$\Pr[|S_\kappa| > 4\log^{a+1} n] = o(n^{-1}).$$

Therefore, with probability $1 - o(n^{-1})$, the size of S_κ is at most $4\log^{a+1} n$.

For (4). We need to bound the number of global edges with one endpoint in S.

For $t \geq t_S$, define $S[t]$ to be the snapshot of S at time step t, and $\partial(S)[t]$ to be the set of edges from $S[t]$ to $\overline{S[t]}$, the complement of $S[t]$. So $\partial(S)[t]$ is in fact the set of global edges of S at time step t and $g_S = |\partial(S)[n]|$. Denote by $D(S)[t]$ the total degree of nodes in (volume of) $S[t]$. In our proof, we first give a recurrence for the expected value of $D(S)[t]$ at any time step $t > t_S$, and then show that $\partial(S)[n]$ is not expectedly too many.

By the construction of G, the recurrence on $D(S)[t]$ can be written as

$$\begin{aligned} E[D(S)[t] \mid D(S)[t-1]] &= D(S)[t-1] + \frac{1}{\log^a t} \cdot \frac{D(S)[t-1]}{2d(t-1)} \cdot d \\ &\quad + \left(1 - \frac{1}{\log^a t}\right) \cdot \frac{2d}{|C_{t-1}|}. \end{aligned} \tag{1}$$

We suppose the event $\mathcal{E}$ that for all $t \geq T_1 = \log^{a+1} n$, $\frac{t}{2\log^a t} \leq |C_t| \leq \frac{2t}{\log^a t}$, which almost surely holds by (1). It also holds for $t \geq T_2$ for sufficiently large n. On this condition,

$$E(D(S)[t] \mid D(S)[t-1], \mathcal{E}) \leq D(S)[t-1] \left[1 + \frac{1}{2(t-1)\log^a t}\right] + \frac{4d\log^a t}{t}. \tag{2}$$

Taking expectation on both sides, we have

$$E(D(S)[t]) \leq E(D(S)[t-1]) \left[1 + \frac{1}{2(t-1)\log^a t}\right] + \frac{4d\log^a t}{t}. \tag{3}$$

Then we analyze this recurrence for the cases of $a \geq 1$ and $a < 1$, respectively.

When $a \geq 1$, since for sufficiently large n and thus for sufficiently large t with $t \geq t_S \geq T_2$,

we have

$$\begin{aligned}
& 9d\log^{a+1}(t+1) - \left[1+\frac{1}{2(t-1)\log^a t}\right]\cdot 9d\log^{a+1} t \\
\geq\ & 9d\log^a t\log\frac{t+1}{t} - \frac{9d\log t}{2(t-1)} \\
\geq\ & \frac{9d\log^a t}{t+1} - \frac{9d\log^a t}{2(t-1)} \\
\geq\ & \frac{4d\log^a t}{t},
\end{aligned} \tag{4}$$

where the second inequality follows from Fact 1. Applying it to Inequality (3), we have

$$E(D(S)[t]) - 9d\log^{a+1}(t+1) \leq \left[1+\frac{1}{2(t-1)\log^a t}\right]\cdot (E(D(S)[t-1]) - 9d\log^{a+1} t).$$

Recursively, we have that

$$E(D(S)[t]) \leq \theta_t \cdot [E(D(S)[t_S]) - 9d\log^{a+1}(t_S+1)] + 9d\log^{a+1}(t+1)$$

holds for all $t_S < t \leq n$, where

$$\theta_t = \prod_{i=t_S+1}^{t}\left[1+\frac{1}{2(i-1)\log^a i}\right].$$

Note that $E(D(S)[t_S]) = d$. So

$$E(D(S)[t]) \leq 9d\log^{a+1}(t+1) - \theta_t \cdot [9d\log^{a+1}(t_S+1) - d]. \tag{5}$$

When $0 < a < 1$, since for sufficiently large n and thus for sufficiently large t,

$$\begin{aligned}
& \left[1+\frac{1}{2(t-1)\log^a t}\right]\cdot 9d\log^{2a} t - 9d\log^{2a}(t+1) \\
=\ & \frac{9d\log^a t}{2(t-1)} - 9d\cdot[\log^{2a}(t+1) - \log^{2a} t] \\
\geq\ & \frac{9d\log^a t}{2(t-1)} - \frac{d\log^a t}{2t} \\
\geq\ & \frac{4d\log^a t}{t},
\end{aligned} \tag{6}$$

where the first inequality follows from the fact that $\log(t+1)-\log t = \log\left(1+\frac{1}{t}\right) \leq \frac{1}{t}$ and $a<1$,

$$\begin{aligned}
\lim_{t\to\infty} \frac{\log^{2a}(t+1)-\log^{2a} t}{\frac{\log^a t}{t}} &= \lim_{t\to\infty} t\cdot\left[\frac{\log^a(t+1)}{\log^a t}-1\right]\cdot(\log^a(t+1)+\log^a t) \\
&\leq \lim_{t\to\infty} t\cdot\left[\frac{\log(t+1)}{\log t}-1\right]\cdot(\log^a(t+1)+\log^a t) \\
&\leq \lim_{t\to\infty} t\cdot\frac{\log(t+1)-\log t}{\log t}\cdot 2\log^a(t+1) \\
&\leq \lim_{t\to\infty} \frac{2\log^a(t+1)}{\log t} = 0.
\end{aligned}$$

Applying Inequality (6) to (3), we have

$$E(D(S)[t]) + 9d\log^{2a}(t+1) \leq \left[1+\frac{1}{2(t-1)\log^a t}\right]\cdot(E(D(S)[t-1]) + 9d\log^{2a} t).$$

Recursively, we have that

$$E(D(S)[t]) \leq \theta_t\cdot[E(D(S)[t_S]) + 9d\log^{2a}(t_S+1)] - 9d\log^{2a}(t+1)$$

holds for all $t_S < t \leq n$, and so

$$E(D(S)[t]) \leq \theta_t\cdot(9d\log^{2a}(t_S+1)+d) - 9d\log^{2a}(t+1). \tag{7}$$

Note that by the construction of G,

$$E(g_S) = \sum_{t=t_S}^{n} \frac{1}{\log^a t}\cdot\frac{E(D(S)[t])}{2(t-1)}\cdot d. \tag{8}$$

Next, we will bound $E(g_S)$ by using Inequalities (5) and (7) for different values of a.

When $a \geq 1$, we have

$$E(g_S) \leq \sum_{t=t_S}^{n} \frac{9d\log^{a+1}(t+1) - \theta_t\cdot[9d\log^{a+1}(t_S+1)-d]}{2(t-1)\log^a t}\cdot d.$$

Since $\theta_t > 1$, for sufficient large n, we have

$$
\begin{aligned}
E(g_S) &\le \sum_{t=t_S}^{n} \frac{9d\log^{a+1}(t+1) - [9d\log^{a+1}(t_S+1) - d]}{2(t-1)\log^a t} \cdot d \\
&= \frac{9d^2}{2}\left[\sum_{t=t_S}^{n} \frac{\log t}{t-1} - \left[\log^{a+1}(t_S+1) - \frac{1}{9}\right]\sum_{t=t_S}^{n}\frac{1}{(t-1)\log^a t}\right] \\
&\le 5d^2 \cdot \left(\int_{t_S}^{n} \frac{\log x}{x}dx - \log^{a+1} t_S \int_{t_S}^{n} \frac{1}{x\log^a x}dx\right).
\end{aligned}
$$

If $a > 1$, then

$$
\begin{aligned}
E(g_S) &\le 5d^2 \cdot \left[\frac{1}{2}(\log^2 n - \log^2 t_S) - \frac{\log^{a+1} t_S}{1-a}(\log^{1-a} n - \log^{1-a} t_S))\right] \\
&= 5d^2\log^2 n \cdot \left[\frac{1}{2} - \left(\frac{1}{2} + \frac{1}{a-1}\right)\left(\frac{\log t_S}{\log n}\right)^2 + \frac{1}{a-1}\left(\frac{\log t_S}{\log n}\right)^{a+1}\right] \\
&= 5d^2\log^2 n \cdot \left[\frac{1}{2} - \frac{a+1}{2(a-1)}\left(1 - \frac{b\log\log n}{\log n}\right)^2 + \frac{1}{a-1}\left(1 - \frac{b\log\log n}{\log n}\right)^{a+1}\right].
\end{aligned}
$$

By Fact 2,

$$
\left(1 - \frac{b\log\log n}{\log n}\right)^{a+1} \le 1 - \frac{(a+1)b\log\log n}{\log n} + \frac{(a+1)ab^2(\log\log n)^2}{2\log^2 n}.
$$

Thus,

$$
\begin{aligned}
E(g_S) &\le 5d^2\log^2 n \cdot \left[\frac{1}{2} - \frac{a+1}{2(a-1)}\left(1 - \frac{2b\log\log n}{\log n} + \frac{b^2(\log\log n)^2}{\log^2 n}\right)\right. \\
&\quad \left. + \frac{1}{a-1}\left(1 - \frac{(a+1)b\log\log n}{\log n} + \frac{(a+1)ab^2(\log\log n)^2}{2\log^2 n}\right)\right] \\
&= \frac{5}{2}d^2b^2(a+1)(\log\log n)^2.
\end{aligned}
$$

(4) (i) follows.

If $a = 1$, then

$$
\begin{aligned}
E(g_S) &\leq 5d^2 \cdot \left(\int_{t_S}^{n} \frac{\log x}{x} dx - \log^2 t_S \int_{t_S}^{n} \frac{1}{x \log x} dx \right) \\
&= 5d^2 \left[\frac{1}{2} (\log^2 n - \log^2 t_S) - \log^2 t_S \cdot (\log\log n - \log\log t_S) \right] \\
&= 5d^2 \left[\frac{1}{2} (\log^2 n - \log^2 t_S) - \log^2 t_S \cdot \log \left(1 + \frac{b \log\log n}{\log n - b \log\log n} \right) \right] \\
&\leq 5d^2 \left[\frac{1}{2} (\log^2 n - \log^2 t_S) - \log^2 t_S \cdot \frac{b \log\log n}{\log n} \right] \\
&= 5d^2 \left[\frac{1}{2} \log^2 n - \frac{1}{2} (\log n - b \log\log n)^2 - (\log n - b \log\log n)^2 \cdot \frac{b \log\log n}{\log n} \right] \\
&= 5d^2 \left[\frac{3}{2} b^2 (\log\log n)^2 - \frac{(b \log\log n)^3}{\log n} \right] \\
&\leq 8d^2 b^2 (\log\log n)^2.
\end{aligned}
$$

(4) (ii) follows.

When $a < 1$, applying Inequality (7) to (8), we have

$$\begin{aligned}
E(g_S) &\le \sum_{t=t_S}^{n} \frac{\theta_t \cdot (9d\log^{2a}(t_S+1)+d) - 9d\log^{2a}(t+1)}{2(t-1)\log^a t} \cdot d \\
&\le \frac{9d^2}{2} \cdot \left[\sum_{t=t_S}^{n} \frac{\theta_n \log^{2a} t_S}{(t-1)\log^a t} - \sum_{t=t_S}^{n} \frac{log^{2a}(t+1)}{(t-1)\log^a t}\right] \\
&= \frac{9d^2}{2} \cdot \left(\theta_n \log^{2a} t_S \cdot \int_{t_S}^{n} \frac{1}{x\log^a x} dx - \int_{t_S}^{n} \frac{log^a x}{x} dx\right) + O\left(\frac{1}{n}\right) \\
&= \frac{9d^2}{2} \cdot \left(\theta_n \log^{2a} t_S \cdot \frac{\log^{1-a} n - \log^{1-a} t_S}{1-a} - \frac{\log^{1+a} n - \log^{1+a} t_S}{1+a}\right) + O\left(\frac{1}{n}\right) \\
&= \frac{9d^2\theta_n}{2(1-a)} \log^{1-a} n \log^{2a} t_S - \frac{9d^2}{2}\left(\frac{\theta_n}{1-a} - \frac{1}{1+a}\right) \log^{1+a} t_S \\
&\quad - \frac{9d^2}{2(1+a)} \log^{1+a} n + O\left(\frac{1}{n}\right) \\
&= \frac{9d^2\theta_n}{2(1-a)} \log^{1+a} n \left(1 - \frac{b\log\log n}{\log n}\right)^{2a} - \frac{9d^2}{2}\left(\frac{\theta_n}{1-a} - \frac{1}{1+a}\right) \log^{1+a} n \\
&\quad \cdot \left(1 - \frac{b\log\log n}{\log n}\right)^{1+a} - \frac{9d^2}{2(1+a)} \log^{1+a} n + O\left(\frac{1}{n}\right)
\end{aligned}$$

$$\begin{aligned}
&= \frac{9d^2\theta_n}{2(1-a)} \log^{1+a} n \cdot \left[1 - \frac{2ab\log\log n}{\log n} + \frac{2a(2a-1)}{2}\left(\frac{b\log\log n}{\log n}\right)^2 \right. \\
&\quad \left. + O\left(\frac{\log\log n}{\log n}\right)^3\right] - \frac{9d^2}{2}\left(\frac{\theta_n}{1-a} - \frac{1}{1+a}\right) \log^{1+a} n \cdot \left[1 - \frac{(1+a)b\log\log n}{\log n}\right. \\
&\quad \left. + \frac{(a+1)a}{2}\left(\frac{b\log\log n}{\log n}\right)^2 + O\left(\frac{\log\log n}{\log n}\right)^3\right] - \frac{9d^2}{2(1+a)} \log^{a+1} n + O\left(\frac{1}{n}\right) \\
&= \left[\frac{9d^2\theta_n}{2(1-a)} - \frac{9d^2}{2}\left(\frac{\theta_n}{1-a} - \frac{1}{1+a}\right) - \frac{9d^2}{2(1+a)}\right] \cdot \log^{1+a} n + \left[-\frac{9d^2}{2(1-a)} \cdot 2ab \right. \\
&\quad \left. + \frac{9d^2}{2}\left(\frac{\theta_n}{1-a} - \frac{1}{1+a}\right) \cdot (1+a)b\right] \cdot \log^a n \log\log n + O\left[\frac{(\log\log n)^2}{\log^{1-a} n}\right] \\
&= \frac{9}{2} d^2 b(\theta_n - 1) \log^a n \log\log n + O\left[\frac{(\log\log n)^2}{\log^{1-a} n}\right].
\end{aligned}$$

To deal with the factor $(\theta_n - 1)$, we need the following lemma.

Lemma 4 *For sufficiently large* n*,*

$$\theta_n - 1 \leq \frac{b \log \log n}{\log^a n}.$$

Note that by the above lemma, for sufficiently large n

$$\begin{aligned} E(g_S) &\leq \frac{9}{2} d^2 b \cdot \frac{b \log \log n}{\log^a n} \log^a n \log \log n + O\left[\frac{(\log \log n)^2}{\log^{1-a} n}\right] \\ &\leq 5 d^2 b^2 (\log \log n)^2, \end{aligned}$$

and hence (4) (iii) follows.

To complete the proof, we prove the lemma, i.e., Lemma 4.

Recall that

$$\theta_n = \prod_{i=t_S+1}^{n} \left[1 + \frac{1}{2(i-1)\log^a i}\right].$$

Then:

$$\begin{aligned} \log \theta_n &= \sum_{i=t_S+1}^{n} \log\left[1 + \frac{1}{2(i-1)\log^a i}\right] \\ &\leq \sum_{i=t_S+1}^{n} \frac{1}{2(i-1)\log^a i} \\ &\leq \frac{1}{2} \int_{t_S}^{n} \frac{1}{x \log^a x} \end{aligned}$$

$$
\begin{aligned}
&= \frac{1}{2(1-a)} \cdot (\log^{1-a} n - \log^{1-a} t_S) \\
&= \frac{\log^{1-a} n}{2(1-a)} \cdot \left[1 - \left(1 - \frac{b \log\log n}{\log n}\right)^{1-a}\right] \\
&= \frac{\log^{1-a} n}{2(1-a)} \cdot \left[(1-a) \cdot \frac{b \log\log n}{\log n} - \frac{(1-a)(-a)}{2} \cdot \left(\frac{b \log\log n}{\log n}\right)^2 \right. \\
&\quad \left. + O\left(\frac{\log\log n}{\log n}\right)^3\right] \\
&= \frac{b \log\log n}{2 \log^a n} + O\left[\frac{(\log\log n)^2}{\log^{1+a} n}\right].
\end{aligned}
$$

Thus, for sufficiently large n, $\log \theta_n \leq \frac{3b \log\log n}{4 \log^a n}$, which implies that

$$
\theta_n \leq (\log n)^{\frac{3b}{4 \log^a n}}.
$$

A key observation is that, for any constant c, by l'Hôpital's rule,

$$
\begin{aligned}
\lim_{n\to\infty} \frac{(\log n)^{\frac{c}{\log^a n}} - 1}{\frac{\log\log n}{\log^a n}} &= \lim_{y\to\infty} \frac{y^{\frac{c}{y^a}} - 1}{\frac{\log y}{y^a}} = \lim_{y\to\infty} \frac{\left(y^{\frac{c}{y^a}} - 1\right)'}{\left(\frac{\log y}{y^a}\right)'} \\
&= \lim_{y\to\infty} \frac{c(1 - a\log y)}{y^{1+a-\frac{c}{y^a}}} \cdot \frac{y^{1+a}}{1 - a\log y} \\
&= \lim_{y\to\infty} c \cdot y^{\frac{c}{y^a}} = \lim_{y\to\infty} c \cdot e^{\frac{c \log y}{y^a}} = c.
\end{aligned}
$$

Thus, for any $\epsilon > 0$, if n is large enough, then

$$
\theta_n - 1 \leq \frac{3b}{4}(1+\epsilon) \cdot \frac{\log\log n}{\log^a n}.
$$

Let $\epsilon = \frac{1}{3}$, then the lemma follows.

This completes the proof of Theorem 1.

Power Law and Holographic Law

In this section, we prove that the degrees of the networks generated by the homophyly/kinship model follow a *power law*, and a *holographic law*. Let G be such a network. We say that a homochromatic set of G is a *natural community* or *community* of G. We will show that there exists a constant β such that almost surely, the degrees of a natural community, the degrees of the induced subgraph of a natural community, and the degrees of the whole network G, all follow a power law with power exponent the same constant β.

Theorem 2 *(Power law and holographic law) For $a \geq 0$ and $d \geq 2$, let $G = (V, E)$ is a network constructed from $\mathcal{H}(n, a, d)$. Then we have:*

(1) For $a = 0$, the degree of nodes in G follows a power law distribution.

(2) For $a > 0$, there exists a constant β satisfying:

(i) The degrees of the induced subgraph of almost all homochromatic sets follow a power law with power exponent β.

(ii) The degrees of nodes of almost all homochromatic sets follow a power law with power exponent β.

(iii) (Power law) Degrees of nodes in G follow a power law with power exponent β.

By Theorem 1 (3), a natural community is small. By Theorem 2, a natural community is interpretable by common attributes of the nodes, the common color here, and almost all the com-

munities have degree patterns similar to that of the whole graph, showing a type of *self-similarity* of networks. (2) (i), (ii) and (2) (iii) of Theorem 2 explore a *holographic law* of the homophyly networks, that the power exponent of the whole network is contained in a natural community of the network.

For (1). If $a = 0$, then the homophyly model degenerates to the classic PA model, and the power law degree distribution with $\beta = 3$ can be obtained by the canonical proof of the PA model[3].

For (2). We prove the following two items together, which proves (2) (i) and (2) (ii), respectively.

(A) For almost every homochromatic set X, the degree distribution of the induced subgraph G_X follows a power law, and

(B) For almost every homochromatic set X, the degrees of nodes in X follow a power law.

(2) (iii) will follow immediately from (B) by observing that the union of several power law distributions with the same power exponent is also a power law distribution.

The proofs of (A) and (B) are similar to the canonical proof of the PA model. Additionally, we will verify that the contribution of degrees of a community from global edges is negligible compared with those from its local edges. So the construction of a homochromatic set basically follows the classic preferential attachment scheme, and the number of global edges created by other seed nodes is negligible. We will realize this idea gradually in the proofs below.

Let $T_3 = (1-\delta_1)n$, where $\delta_1 = \frac{1}{\log^{a/2} n}$. Let X denote a homochromatic set of a fixed color and t_X be the time step at which X is created. Suppose that $T_2 \le t_X \le T_3$. By Theorem 1, since there are $\Theta(n/\log^a n)$ homochromatic sets in G_n and each of them has size $O(\log^{a+1} n)$ with extremely high probability, it is easy to show that if $b > 2a+1$ for $T_2 = n/\log^b n$, then almost all homochromatic sets are created in the time interval (T_2, T_3) with probability $1-o(1)$. So to prove (A) and (B), we only analyze the homochromatic sets created in this time interval.

For positive integers s and k, define $A_{s,k}$ to be the number of nodes of degree k in X when $|X|$ reaches s, $B_{s,k}$ the number of nodes of degree k in the induced subgraph of X when $|X|$ reaches s, and $g_{s,k}$ the number of global edges associated with the nodes in X of degree k in the induced subgraph of X when $|X|$ reaches s. Obviously, we have $A_{s,k} = B_{s,k} + g_{s,k}$, $A_{1,d} = 1$, $A_{1,k} = 0$ for all $k > d$, and $B_{1,k} = 0$ for all k. Then we establish the recurrence formula for the expectation of both $A_{s,k}$ and $B_{s,k}$.

Define $T(s)$ to be the time step at which the size of X becomes to be s, and s' to be the number of global edges connecting X in the case that $|X| = s$ (note that probably at several consecutive time steps, $|X|$ keeps s). We consider the time interval $(T(s-1), T(s))$.

By Theorem 1, since $T_1 \le T_2 \le t_X \le T_3$, the size of X at time step n is expected to be $E(|X|) = \Theta(\log^{a+1} n - \log^{a+1} t_S) = \Omega\left(\log^a n \cdot \log \frac{1}{1-\delta_1}\right) = \Omega(\log^{a/2} n)$, and the number of global edges is expected to be $O(\log\log n)^2 = o(E(|X|))$. Thus, in the time interval $(T(s-1), T(s))$, the number of global edges that link to a node in X is $o(s^{-1})$.

For $s > 1$ and $k > d$, we have

$$\begin{aligned} E(A_{s,k}) &= A_{s-1,k} \cdot \left[1 - \frac{kd}{2d(s-1)+s'} - o\left(\frac{1}{s}\right)\right] \\ &\quad + A_{s-1,k-1} \cdot \left[\frac{(k-1)d}{2d(s-1)+s'} + o\left(\frac{1}{s}\right)\right] + O\left(\frac{1}{s^2}\right), \end{aligned}$$

where the error terms caused by the case that more than one edge joins to a single node are absorbed in the term $O(1/s^2)$. Taking expectations on both sides, we have

$$\begin{aligned} E(A_{s,k}) &= E(A_{s-1,k}) \cdot \left[1 - \frac{k}{2(s-1)+s'/d} - o\left(\frac{1}{s}\right)\right] \\ &\quad + E(A_{s-1,k-1}) \cdot \left[\frac{(k-1)}{2(s-1)+s'/d} + o\left(\frac{1}{s}\right)\right] + O\left(\frac{1}{s^2}\right), \end{aligned} \tag{9}$$

When $k = d$,

$$E(A_{s,d}) = E(A_{s-1,d}) \cdot \left[1 - \frac{d}{2(s-1)+s'/d} + o\left(\frac{1}{s}\right)\right] + 1 + O\left(\frac{1}{s^2}\right). \tag{10}$$

Similarly, for $s > 1$ and $k > d$,

$$E(B_{s,k}) = B_{s-1,k} - \frac{d \cdot (kB_{s-1,k} + g_{s-1,k})}{2d(s-1)+s'} + \frac{d \cdot [(k-1)B_{s-1,k-1} + g_{s-1,k-1}]}{2d(s-1)+s'} + O\left(\frac{1}{s^2}\right).$$

Taking expectations on both sides, we have

$$\begin{aligned} E(B_{s,k}) &= E(B_{s-1,k}) \cdot \left[1 - \frac{kd}{2d(s-1)+s'}\right] + E(B_{s-1,k-1}) \cdot \frac{(k-1)d}{2d(s-1)+s'} \\ &\quad + \frac{E(g_{s-1,k-1} - g_{s-1,k})}{2d(s-1)+s'} + O\left(\frac{1}{s^2}\right). \end{aligned} \tag{11}$$

When $k = d$,

$$\begin{aligned} E(B_{s,d}) &= B_{s-1,d} - \frac{d \cdot (dB_{s-1,d} + g_{s-1,d})}{2d(s-1)+s'} + 1 + O\left(\frac{1}{s^2}\right) \\ &= B_{s-1,d} \cdot \left[1 - \frac{d}{2(s-1)+s'/d}\right] + \left[1 - \frac{g_{s-1,d}}{2(s-1)+s'/d}\right] + O\left(\frac{1}{s^2}\right), \end{aligned}$$

and

$$E(B_{s,d}) = E(B_{s-1,d}) \cdot \left[1 - \frac{d}{2(s-1) + s'/d}\right] + \left[1 - \frac{E(g_{s-1,d})}{2(s-1) + s'/d}\right] + O\left(\frac{1}{s^2}\right). \quad (12)$$

To solve these recurrences, we introduce the following lemma that is used in the canonical proof of the preferential attachment model.

Lemma 5 *(Lemma 3.1[2]) Suppose that a sequence* $\{a_s\}$ *satisfies the recurrence relation*

$$a_{s+1} = \left(1 - \frac{b_s}{s + s_1}\right) a_s + c_s \ \ \textit{for} \ \ s \geq s_0,$$

where the sequences $\{b_s\}, \{c_s\}$ *satisfy* $\lim_{s\to\infty} b_s = b > 0$ *and* $\lim_{s\to\infty} c_s = c$ *respectively. Then the limit of* $\frac{a_s}{s}$ *exists and*

$$\lim_{s\to\infty} \frac{a_s}{s} = \frac{c}{1+b}.$$

Note that since $|X|$ goes to infinity as $n \to \infty$. By Theorem 1, the terms s'/d in equalities (9) and (10) are comparatively negligible. Since $g_{s,k}$, for each k, is expected to be $o(E(|X|))$, the terms $\frac{E(g_{s-1,k-1} - g_{s-1,k})}{2d(s-1)+s'}$ and $\frac{E(g_{s-1,d})}{2d(s-1)+s'}$ in equalities (11) and (12) are also comparatively negligible. By Lemma 5, $\frac{E(A_{s,k})}{s}$ and $\frac{E(B_{s,k})}{s}$ must have the same limit as t goes to infinity. Thus we will only give the proof of the power law distribution for $E(A_{s,k})$, which also holds for $E(B_{s,k})$.

Denote by $S_k = \lim_{t\to\infty} \frac{E(A_{s,k})}{s}$ for $k \geq d$. In the case of $k = d$, we apply Lemma 5 with $b_s = d/2 + o(1)$, $c_s = 1 + O(1/s^2)$, $s_1 = -1$, and get

$$S_d = \lim_{s\to\infty} \frac{E(A_{s,d})}{t} = \frac{1}{1 + \frac{d}{2}} = \frac{2}{2+d}.$$

For $k > d$, assume that we already have $S_{k-1} = \lim_{t\to\infty} \frac{E(A_{s,k-1})}{t}$. Applying Lemma 5 again with $b_s = k/2 + o(1)$, $c_s = \frac{E(A_{s-1,k-1})}{s-1} \cdot \frac{k-1}{2} + O(1/s^2)$, $s_1 = -1$, we get

$$S_k = \lim_{t\to\infty} \frac{E(A_{s,k})}{s} = \frac{S_{k-1} \cdot \frac{k-1}{2}}{1 + \frac{k}{2}} = S_{k-1} \cdot \frac{k-1}{k+2}.$$

Thus recurrently, we have

$$S_k = S_d \cdot \frac{(d+2)!(k-1)!}{(d-1)!(k+2)!} = \frac{2d(d+1)}{k(k+1)(k+2)}. \tag{13}$$

This implies

$$|E(A_{s,k}) - S_k \cdot s| = o(s),$$

and thus

$$E(A_{s,k}) = (1 + o(1))k^{-3}s. \tag{14}$$

Since $s = \omega(1)$ goes to infinity as $n \to \infty$, $E(A_{s,k}) \propto k^{-3}$. For the same reason, $E(B_{s,k}) \propto k^{-3}$. This proves (A) and (B), and also completes the proof of (2) (i) and (2) (ii).

For (2) (iii), a key observation is that the union of several power law distributions is also a power law distribution if the power exponents are equal. We will give the same explicit expression of the expectation of the number of degree k nodes by combining those for the homochromatic sets, leading to a similar power law distribution.

To prove the power law degree distribution of the whole graph, we take the union of distributions of all homochromatic sets. Suppose that G_n has m homochromatic sets that are created in

time interval (T_2, T_3). For $i = 1, \ldots, m$, let M_i be the size of the i-th homochromatic set and $N^{(i)}_{s,k}$ denote the number of nodes of degree k when the i-th set has size s. For each i, we have

$$\lim_{n\to\infty} \frac{E(N^{(i)}_{M_i,k})}{M_i} = S_k.$$

Hence,

$$\lim_{n\to\infty} \frac{E(\sum_{i=1}^m N^{(i)}_{M_i,k})}{\sum_{i=1}^m M_i} = S_k.$$

Let M_0 denote the size of the union of all other homochromatic sets and $N^{(0)}_{s,k}$ denote the number of nodes of degree k in this union when it has size s. By Theorem 1, we know that $E(M_0) = o(n)$. Since the color of each node is chosen independently, by the Chernoff bound, $M_0 = o(n)$ holds with probability $1 - o(1)$. Define $N_{t,k}$ to be the number of nodes of degree k in G_t. Then we have

$$\lim_{n\to\infty} \frac{E(N_{n,k})}{n} = \lim_{n\to\infty} \frac{E(\sum_{i=0}^m N^{(i)}_{M_i,k})}{\sum_{i=0}^m M_i}.$$

For M_0, we have that

$$\lim_{n\to\infty} \frac{M_0}{\sum_{i=1}^m M_i} = \lim_{n\to\infty} \frac{M_0}{n - M_0} = 0$$

and

$$\lim_{n\to\infty} \frac{E(N^{(0)}_{M_0,k})}{n} \le \lim_{n\to\infty} \frac{M_0}{n} = 0$$

hold with probability $1 - o(1)$. So

$$\lim_{n\to\infty} \frac{E(N_{n,k})}{n} = \lim_{n\to\infty} \frac{E(\sum_{i=1}^m N^{(i)}_{M_i,k})}{\sum_{i=1}^m M_i} = S_k.$$

This implies

$$|E(N_{n,k}) - S_k \cdot n| = o(n),$$

and thus,

$$E(N_{n,k}) = (1 + o(1))k^{-3}n,$$

and $E(N_{n,k}) \propto k^{-3}$. (2) (iii) follows.

This completes the proof of Theorem 2.

Small Diameter Property

In this section, we show that the diameters of the networks generated from the homophyly/kinship model is small.

Theorem 3 *(Small diameter property) For $a \geq 0$, let $G = (V, E)$ be a graph generated from $\mathcal{H}(n, a, d)$. Then the following properties hold:*

(1) For $a = 0$, with probability $1 - o(1)$, the diameter of G is $O(\log n)$.

(2) For $a > 0$, with probability $1 - o(1)$, the diameter of G is $O(\log^{a+2} n)$.

We remark that the small world phenomenon consists of two properties, the first is the small diameter property, and the second is the clustering effect. Our theorem shows the first. For the second property, it is easy to see that if we add some local rules in the creation of local edges, i.e., the edges between nodes of the same color, then the clustering effect will be significantly amplified. It is important that this kind of modifications will never change all the other properties

of the graphs. The idea of introducing local rules in creation of local edges here is interesting. It has been a hard open problem to combine the two properties of the small world phenomenon in modeling, as commented by Chung and Lu in [2]. To our knowledge, the only known theoretical results are on grid-like graphs by Kleinberg[4], leading to local algorithms for finding short paths. It would be an interesting problem to better fit our model by introducing some local rules to generate graphs with both small diameter property and the clustering effect and to allow local algorithms for communications in the graphs.

Now we turn to prove Theorem 3.

For (1). When $a = 0$, the homophyly/kinship model is the classic PA model, and the diameter has been proved to be $O(\log n)$ with probability $1 - o(1)$ by Bollobás and Riordan [5].

For (2). Let $a > 0$ and G be a graph in $\mathcal{H}(n, a, d)$. We consider the hierarchical structure of G. Let G' be the graph obtained from G by merging all non-seed nodes of each homochromatic set to the corresponding seed node. Hence the nodes of G' are the seed nodes of G, and the edges of G' are the global edges of G. Let G'' be the graph obtained from G by deleting all global edges. In so doing, G'' consists of the isolated homochromatic sets.

Given two nodes u and v in G, suppose that u_0 and v_0 are the seed nodes of colors as the same as that of u and v respectively. By definition, u_0 and v_0 are nodes in G'. We choose a path $P_{u,v}$ connecting two nodes u, v in G as follows. We consider two cases.

Case 1. u and v share the same color.

Let X be the homochromatic set of u. In this case, we define $P_{u,v}$ to be the shortest path between u and v in the induced subgraph of the natural community X.

Case 2. Otherwise. Then

Suppose that $u_0, u_1, \cdots, u_l = v_0$ is a shortest path in G', where each u_i is a seed node of G. For each i, let X_i be the set of nodes sharing the same color as that of u_i.

Let P_{u,u_0} and $P_{v_0,v}$ be the shortest paths between u and u_0 in X_0, and between v_0 and v in X_l, respectively. For every i with $0 \leq i < l$, let $P_{u_i,u_{i+1}}$ be the path consisting of a shortest path in X_i, a shortest path in X_{i+1} and an edge between X_i and X_{i+1}. We define $P_{u,v}$ to be the path consisting the paths P_{u,u_0}, P_{u_0,u_1}, $\cdots$, $P_{u_{l-1},u_l}, p_{u_l,v}$. By definition, there are l edges in G' are used in the path $P_{u,v}$ and $P_{u,v}$ consists alternately of paths composed by local and global edges..

To estimate the number l, we recall a known result on random recursive trees. A random recursive tree is constructed by stages. At each stage, a new vertex is created which links to an earlier node randomly. If each node is picked uniformly, then we call it a uniform recursive tree. We use a result of Pittel in [6], saying that the height of a uniform recursive tree of size n is $O(\log n)$ with high probability.

Lemma 6 *(Recursive tree lemma[6]) With probability* $1 - o(1)$*, the height of a uniform recursive tree of size* n *is asymptotic to* $e \log n$*, where* e *is the natural logarithm.*

Consider G' as a union of d recursive trees. Notice that the earlier created homochromatic sets in G have larger expected volumes than that of the communities created later. So with higher probability than the uniform recursive tree, the height of a recursive tree in G' is at most $e \log |C_n|$, where $|C_n|$ is the number of colors in G and is also the number of nodes in G'. This means that with probability $1 - o(1)$, the diameter of G' is an upper bounded by $2e \log |C_n| = O(\log n)$. Therefore the number l in the definition of $P_{u,v}$ is at most $O(\log n)$.

On the other hand, by Theorem 1, with probability $1 - o(1)$, every homochromatic set has a size upper bounded by $O(\log^{a+1} n)$. Hence, for every i, the length of $P_{u_i,u_{i+1}}$ is at most $O(\log^{a+1} n)$.

Therefore, with probability $1 - o(1)$, the diameter of G is upper bounded by $O(\log^{a+1} n) \cdot O(\log n) = O(\log^{a+2} n)$. (2) follows.

This completes the proof of Theorem 3.

Since the diameter of a PA network is expected to be logarithmic of its size, the diameter of each homochromatic set is expected to be $O(\log \log n)$. This implies that the expected diameter of a homophyly network is $O(\log n \log \log n)$.

However, for concentration probability, our result in Theorem 3 is only a rough upper bound $O(\log^{a+2} n)$ for the diameter. It is an interesting open question is to prove or disprove the concentration result of diameter $O(\log n \log \log n)$ for the homophyly/kinship networks.

Generally speaking, there are interesting problems left open by our model. The first is to modify the model to generate networks with both small diameter property and clustering effect simultaneously. The second is to develop a theoretical approach to algorithmic small world phenomenon, for which it would be very interesting to design algorithms to find short paths on networks of small diameters in time complexity polynomial of the diameters.

Conductance Law

Given a graph $G = (V, E)$, and a subset S of V, the conductance of S in G is given by

$$\Phi(S) = \frac{|E(S, \bar{S})|}{\min\{\mathrm{vol}(S), \mathrm{vol}(\bar{S})\}}, \tag{15}$$

where $E(S, \bar{S})$ is the set of edges with one endpoint in S and the other in the complement of S, i.e. $\bar{S}$, and $\mathrm{vol}(X)$ is the sum of degrees d_x for all $x \in X$. The conductance of G is defined to be the minimum of $\Phi(S)$ over all subsets S's, that is,

$$\Phi(G) = \min_{S \subset V}\{\Phi(S)\}. \tag{16}$$

Then we extend the definition of conductance of a set to a class of sets.

Definition 2 *(Structure conductance by a partition) Let G be a graph, and $\mathcal{P} = \{X_1, X_2, \cdots, X_L\}$ be a partition of vertices of G such that $vol(X_i) \leq vol(G)/2 = m$ for each i. Then we define the structure conductance of G by $\mathcal{P}$ as follows.*

$$\theta^{\mathcal{P}}(G) = \frac{1}{n}\sum_{j=1}^{L} |X_j| \cdot \Phi(X_j). \tag{17}$$

$\theta^{\mathcal{P}}(G)$ is in fact the weighted average of conductance for each module of partition $\mathcal{P}$.

Definition 3 *(Structure conductance) Given a graph G, we define the structure conductance of G by*

$$\theta(G) = \min_{\mathcal{P}}\{\theta^{\mathcal{P}}(G)\}. \tag{18}$$

The motivations of the structure conductance of a network are:

- Intuitively speaking, if a set $X \subset V$ has a small conductance, then the internal links of X are strong, and the external links of X are weak. This exactly captures the common convention of quality communities.
- By definition, if $\theta(G)$ is small, then there is a well-defined community structure $\mathcal{P}$ in G. It is clear that a good community structure indicated by low structure conductance requires a mass of nodes belonging to high-quality communities.

In this section, we investigate the structure conductance of the networks generated by the homophyly/kinship model with different values of the homophyly or affinity exponent a. We will

establish a phase transition theorem of structure conductance and the structure conductance minimization principle of the existence of well-defined structures of power law networks. Precisely, we have:

Theorem 4 *(Phase transition of structure conductance, and conductance minimization principle) For $a \geq 0$, $d \geq 2$, let $G = (V, E)$ be a graph generated from $\mathcal{H}(n, a, d)$. We have*

(1) If $a = 0$, then there exists a positive constant α depending only on d such that, with probability $1 - o(1)$, $\theta(G) \geq \alpha$.

(2) If $0 < a \leq 1$, then for almost every homochromatic set S, the expected conductance of S is $O\left(\frac{(\log\log n)^2}{\log^{a/2} n}\right)$, and $E(\theta(G)) = o(1)$.

(3) If $a > 1$, then for $\beta = \frac{a-1}{4(a+1)}$, with probability $1 - o(1)$, for almost every homochromatic set S, the conductance of S, $\Phi(S) = O\left(\frac{1}{|S|^\beta}\right)$. Moreover, with probability $1 - o(1)$, $\theta(G) = o(1)$.

Theorem 4 implies that the structure conductance of networks generated by the homophyly/kinship model decreases as a increases. Furthermore, the structure conductance of the networks decreases in three interesting ways. The results for $a = 0$ and $a > 1$ hold almost surely, and go to the opposite extremes. However for $0 < a \leq 1$, we are just able to prove the expectation result that, the expected structure conductance of network G is $o(1)$. Although the result also implies that even if $0 < a \leq 1$, with high probability, the structure conductance of the networks generated by the

homophyly/kinship model is as small as $o(1)$, it could be possible that, with a non-negligible probability, the structure conductances of some networks of the model are larger than some constant $\alpha > 0$. It is interesting to prove or disprove the concentration result of the structure conductance for the case of $0 < a \leq 1$. Here we leave it as an open question.

Nevertheless the results in Theorem 4 demonstrate that structure conductance minimization is a principle of the existence of community structures of power law networks.

(Prof of Theorem 4) For (1). If $a = 0$, then the homophyly/kinship model degenerates to the PA model. It has been shown that, with probability $1 - o(1)$, networks generated by the PA model are expanders, whose structure conductances has been proved to be at least a constant α depending on d (?, Theorem 1). Since for any partition $\mathcal{P}$ of V, $\theta^{\mathcal{P}}(G)$ is the weighted average over all modules of $\mathcal{P}$ and $\theta^{\mathcal{P}}(G) \geq \alpha$. This implies that $\theta(G) \geq \alpha$.

For (2). Recall that in the proof of the power law, we have shown that if $T_2 \leq t_S \leq T_3$, where $T_2 = \frac{n}{\log^b n}$, $b > 2a+1$, $T_3 = (1-\delta_1)n$, $\delta_1 = \frac{1}{\log^{a/2} n}$, then with probability $1 - o(1)$, almost all the homochromatic sets are created in the time interval (T_2, T_3). So we only have to show that every such homochromatic set, denoted by S, has expected conductance $O\left(\frac{\log \log n}{\log^{a/2} n}\right)$.

Let $0 < a \leq 1$. By Theorem 1, if $t_S \geq T_2$, then the expected number of global edges $E(g_S) = O(\log \log n)^2$. On the other hand, if $t_S \leq T_3$, then the size of S is expected to be $E(|S|) = \Theta(\log^{a+1} n - \log^{a+1} t_S) = \Omega\left(\log^a n \cdot \log \frac{1}{1-\delta_1}\right) = \Omega(\log^{a/2} n)$, and the expected volume of S is also $\Omega(\log^{a/2} n)$. By the Chernoff bound, the results above hold with probability $1 - o(1)$. Hence,

$E(\Phi(S)) = O\left(\frac{(\log\log n)^2}{\log^{a/2} n}\right)$.

To show that $E(\theta(G)) = o(1)$, we only have to show that in the case of a specific partition $\mathcal{P}_1$ in G, $\theta^{\mathcal{P}_1}(G) = o(1)$. We define $\mathcal{P}_1$ by colors such that each homochromatic set S satisfying $T_2 \le t_S \le T_3$ is a module of $\mathcal{P}_1$ and the rest nodes form the last module of $\mathcal{P}_1$. Since $b > 2a+1$ and each homochromatic set has an expected size $O(\log^{a+1} n)$, the size of the last module of $\mathcal{P}_1$ is expected to be at most $O(\log^{a+1} n) \cdot T_2 + \delta_1 n = o(n)$. Thus

$$E(\theta(G)) \le E(\theta^{\mathcal{P}_1}(G)) = O\left(\frac{(\log\log n)^2}{\log^{a/2} n}\right) \cdot (1 - o(1)) + o(1) = o(1).$$

For (3). Assume $a > 1$. Let $T_4 = (1-\delta_2)n$, where $\delta_2 = \frac{1}{\log^{(a-1)/2}} n$. By a similar argument to that for (2) above, we have that for $b = a+2$, almost all the homochromatic sets are created in the time interval (T_2, T_4). We will show that, with probability $1 - o(1)$, every homochromatic set S with $T_2 \le t_S \le T_4$ has conductance $O\left(\frac{1}{|S|^\beta}\right)$.

By the proof of Theorem 1, we can establish an upper bound for the volume of S. We will show a concentration result for the event that the volume of S is $O(\log^{a+1} n)$.

Recall that $D(S)[t]$ is the volume of S at time step t. We have:

Lemma 7 *(Degree of communities lemma) With probability* $1 - o(1)$, *for any homochromatic set* S *created at time* $t_S \ge T_2$, $D(S)[n] = O(\log^{a+1} n)$ *holds.*

We only have to show that for a fixed S with $t_S \ge T_2$, $D_n(S)[n] = O(\log^{a+1} n)$ holds with

probability $1 - o(n^{-1})$. Then the lemma follows from the union bound. We assume the worst case that S is created at time step $t_S = T_2$. In the proof of Theorem 1, we know that the recurrence on $D(S)[t]$ can be written as

$$\begin{aligned} E[D(S)[t] \mid D(S)[t-1]] &= D(S)[t-1] + \frac{1}{\log^a t} \cdot \frac{D(S)[t-1]}{2d(t-1)} \cdot d \\ &\quad + \left(1 - \frac{1}{\log^a t}\right) \cdot \frac{2d}{|C_{t-1}|}, \end{aligned}$$

and thus

$$E(D(S)[t] \mid D(S)[t-1], \mathcal{E}) \leq D(S)[t-1] \left[1 + \frac{1}{2(t-1)\log^a t}\right] + \frac{4d \log^a t}{t}.$$

These are the Inequalities (1) and (2), respectively. Applying Inequality (4) to (2), we have

$$\begin{aligned} & E(D(S)[t] \mid D(S)[t-1], \mathcal{E}) - 9d \log^{a+1}(t+1) \\ \leq \; & \left[1 + \frac{1}{2(t-1)\log^a t}\right] \cdot (D(S)[t-1] - 9d \log^{a+1} t). \end{aligned}$$

Recall that

$$\theta_t = \prod_{i=t_S+1}^{t} \left[1 + \frac{1}{2(i-1)\log^a i}\right].$$

Define $X[t] = \frac{D(S)[t] - 9d \log^{a+1}(t+1)}{\theta_t}$. Then

$$E[X[t] \mid X[t-1], \mathcal{E}] \leq X[t-1].$$

Note that

$$X[t] - E[X[t] \mid X[t-1], \mathcal{E}] = \frac{D(S)[t] - E[D(S)[t] \mid D(S)[t-1], E]}{\theta_t} \leq 2d.$$

Since

$$D(S)[t] - D(S)[t-1] \leq 2d,$$

we have, for sufficiently large n (and so sufficiently large t),

$$\begin{aligned}
\mathrm{Var}[X[t] \mid X[t-1], \mathcal{E}] &= E[(X[t] - E(X[t]|X[t-1], \mathcal{E}))^2] \\
&= \frac{1}{\theta_t^2} E[(D(S)[t] - E(D(S)[t] \mid D(S)[t-1], \mathcal{E}))^2] \\
&\leq \frac{1}{\theta_t^2} E[(D(S)[t] - D(S)[t-1])^2 | D(S)[t-1], \mathcal{E}] \\
&\leq \frac{2d}{\theta_t^2} E[D(S)[t] - D(S)[t-1] \mid D(S)[t-1], \mathcal{E}] \\
&\leq \frac{2d}{\theta_t^2} \left[\frac{4d \log^a t}{t} + \frac{D(S)[t-1]}{2(t-1)\log^a t} \right] \\
&= \frac{8d^2 \log^a t}{t\theta_t^2} + \frac{d}{(t-1)\theta_t \log^a t} \cdot \frac{D(S)[t-1]}{\theta_t} \\
&\leq \frac{8d^2 \log^a t}{t\theta_t^2} + \frac{9d^2 \log^{a+1} t}{(t-1)\theta_t^2 \log^a t} + \frac{dX[t-1]}{(t-1)\theta_t \log^a t} \\
&\leq \frac{10d^2 \log^a t}{t\theta_t^2} + \frac{dX[t-1]}{(t-1)\theta_t \log^a t}.
\end{aligned}$$

Since θ_t can be bounded as

$$\theta_t \sim \exp\left\{ \sum_{i=T_2+1}^{t} \frac{1}{2(i-1)\log^a i} \right\} \in \left[\left(\frac{t}{T_2}\right)^{\frac{1}{2\log^a n}}, \left(\frac{t}{T_2}\right)^{\frac{1}{2\log^a T_2}} \right],$$

we have

$$\sum_{i=T_2+1}^{t} \frac{10d^2 \log^a i}{i\theta_i^2} \leq 10d^2 \log^a n \int_{T_2}^{t} \frac{1}{x} \cdot \left(\frac{T_2}{x}\right)^{\frac{1}{\log^a n}} dx \leq 10d^2 \log^a n \cdot \log n = 10d^2 \log^{a+1} n,$$

and

$$\sum_{i=T_2+1}^{t} \frac{1}{(i-1)\theta_i \log^a i} \leq \frac{2}{\log^a T_2} \int_{T_2}^{t} \frac{T_2^{\frac{1}{2\log n}}}{x \cdot x^{\frac{1}{2\log n}}} dx \leq \frac{2\log n}{\log^a T_2}.$$

Here we can assume that $X[t]$ is non-negative, which means that $D(S)[t] \geq 10\log^{a+1}(t+1)$, because otherwise, $D(S)[n]$ will be smaller and the event in the lemma holds with higher

probability. Let $\lambda = 20\log^{a+1} n$. By Lemma 3,

$$\begin{aligned} & \Pr[X[t] = \omega(\log^{a+1} n)] \le \Pr[X[t] \ge X[T_2] + \lambda] \\ \le & \exp\left\{ -\frac{\lambda^2}{2(10d^2\log^{a+1} n + (2\log n/\log^a T_2)\lambda + d\lambda/3)} \right\} + O(n^{-2}) = O(n^{-2}). \end{aligned}$$

This implies that $D(S)[n] = O(\log^{a+1} n)$ holds with probability $1 - O(n^{-2})$.

Then we consider the number of global edges associated with S with $T_2 \le t_S \le T_4$. Suppose the event, denoted by $\mathcal{F}$, that for any $t \ge t_S$, $D(S)[t] = O(\log^{a+1} n)$, which holds with probability $1 - o(n^{-1})$ by the proof Lemma 7. For each $t \ge t_S$, we define a random variable X_t to be the number of global edges that connect S at time t. We have

$$E(X_t|\mathcal{F}) = d \cdot \frac{1}{\log^a t} \cdot \frac{D(S)[t-1]}{2d(t-1)} \le \frac{\log^{1+\epsilon} n}{2(t-1)},$$

for arbitrarily small positive ϵ. Then

$$E\left(\sum_{t=t_S}^{n} X_t|\mathcal{F}\right) \le \left(\log^{1+\epsilon} n\right) \cdot \sum_{t=t_S}^{n} \frac{1}{2(t-1)} \le a(\log^{1+\epsilon} n)(\log\log n).$$

By the Chernoff bound (conditioned on the event $\mathcal{F}$), for sufficiently large n,

$$\Pr\left[\sum_{t=t_S}^{n} X_t \ge 2a(\log^{1+\epsilon} n)(\log\log n)|\mathcal{F}\right] \le n^{-2}.$$

That is, conditioned on the event $\mathcal{F}$, with probability at least $1 - n^{-2}$, the total number of global edges joining S is upper bounded by $2a(\log^{1+\epsilon} n)(\log\log n)$. Since $\mathcal{F}$ happens with probability $1 - o(n^{-1})$, we have

$$\Pr\left[\sum_{t=t_S}^{n} X_t \ge 2a(\log^{1+\epsilon} n)(\log\log n)\right] = o(n^{-1}).$$

On the other hand, it is easy to show that when $a > 1$, with probability $1 - o(1)$, every such S (satisfying $t_S \in [T_2, T_4]$) has a size $\Omega(\log^{\frac{a+1}{2}} n)$, and so a volume $\Omega(\log^{\frac{a+1}{2}} n)$. Let $0 < \epsilon < \frac{a-1}{4}$. Then, with probability $1 - o(1)$, for each such S,

$$\Phi(S) = O\left(\frac{2a(\log^{1+\epsilon} n)(\log\log n)}{\log^{(a+1)/2} n}\right) \leq O\left(\log^{-\frac{a-1}{4}} n\right) \leq O\left(|S|^{-\frac{a-1}{4(a+1)}}\right).$$

To show that $\theta(G) = o(1)$ holds with probability $1 - o(1)$, as we have done in the proof of (2), we define a partition $\mathcal{P}_2$ and show that $\theta^{\mathcal{P}_2}(G) = o(1)$ with high probability. We define $\mathcal{P}_2$ such that each homochromatic set S satisfying $T_2 \leq t_S \leq T_4$ is a module of $\mathcal{P}_2$ and the rest nodes form the last module of $\mathcal{P}_2$. In fact, by Lemma 7, with probability $1 - o(1)$, the total number of nodes belonging to the homochromatic sets which appear before time T_2 or after T_4, that is the size of the last module of $\mathcal{P}_2$, is at most $O(\log^{a+1} n) \cdot \frac{n}{\log^{a+2} n} + \frac{n}{\log^{(a-1)/2} n} = o(n)$ for constant $a > 1$. Therefore, $1 - o(1)$ fraction of nodes of G belongs to a homochromatic set S of conductance bounded by $O\left(|S|^{-\frac{a-1}{4(a+1)}}\right) = o(1)$. Thus with probability $1 - o(1)$,

$$\theta(G) \leq \theta^{\mathcal{P}_2}(G) = o(1)(1 - o(1)) + o(1) = o(1).$$

This completes the proof of Theorem 4.

The results and proofs of Theorem 4 imply that if $a = 0$, then almost surely the networks of the homophyly/kinship model simply fail to have conductance-based community structure, that if $0 < a \leq 1$, then the networks of the homophyly model may have a conductance-based community structure, but the conductance-based community structures are non-robust, and that if $a > 1$, then

almost surely, the networks of the homophyly/kinship model have a well-defined conductance-based community structure.

It is interesting to notice that Theorem 4 (1) and (3) hold with probability $1 - o(1)$, but Theorem 4 (2) is only an expectation result. This leads to some interesting open questions: Whether or not the result in Theorem 4 (2) can be strengthened to a concentration result? Is structure conductance a robust measure for characterizing the structures or complexity of networks?

Degree priority principle

Let $G = (V, E)$ be a network generated by our homophyly/kinship model. By the construction of G, every node in V is defined a color, interpreted as attribute of the node. On the other hand, a homochromatic set is interpreted as a natural community or module of the network G. According to this understanding, for a node $v \in V$, we are interested not only the degree of v in G, but also the degree of v that is contributed by a specific natural community, Y say, that is, the number of edges between v and nodes in Y, where Y is a homochromatic set of G.

This new feature allows us to refine the degrees of nodes to the j-th degree of node v for various j's, instead of the classic degree of nodes. We notice that in classic graphs and networks, degrees of nodes have already been the basic elements. Our notions refined the fundamental elements of graphs and networks. This refined notion allows us to develop new theory and analyses of networks by a more fundamental approach.

We now look at the new definitions of the j-th degree of nodes for colored graphs.

Definition 4 *Let $G = (V, E)$ be a colored graph. Given a node $v \in V$,*

(1) We define the length of degrees of v to be the number of colors of all the neighbors of v, written by $l(v)$.

Suppose that $Y_1, Y_2, \cdots, Y_l$ are all the homochromatic sets of neighbors of v.

For each $j \in \{1, 2, \cdots, l(v)\}$, let $d_j(v)$ be the number of edges of the form (v, y) for $y \in Y_j$.

Suppose that $d_1(v) \geq d_2(v) \geq \cdots \geq d_l(v)$ (ties break arbitrarily).

(2) Then for each j, we say that $d_j(v)$ is the j-th degree of v.

(3) The degree of v is hence $d(v) = \Sigma_j d_j(v)$.

By Definition 4, we know that orderings are keen to our notion of degrees of node. This is the remarkable difference between our notion of degrees and the classic degrees of nodes. Clearly, orderings of our degrees follow some laws. As a matter of fact, we are able to establish the following *degree priority principle*.

Theorem 5 *(Degree Priority Principle) Let $G = (V, E)$ be a network generated from $\mathcal{H}(n, a, d)$, where $a > 0$ and $d \geq 2$. Then with probability $1 - o(1)$, for almost all nodes v, the following properties hold.*

(1) (First degree property) The first degree of v, $d_1(v)$ is the number of neighbors of v that share the same color as v.

(2) (Second degree property) The second degree of v is bounded by a constant, i.e., $d_2(v) = O(1)$.

(3) (The length of degree)

(a) The length of degrees of v is expected to be $O(\log \log n)$.

(b) If $a > 1$, then the length of degrees of v is $O(\log^{1+\epsilon} n)$.

(4) If v is a seed node, then the first degree of v, $d_1(v)$ is at least $\Omega(\log^{\gamma} n)$ for $\gamma = \frac{a}{4}$.

This principle explores some interesting properties which seem significant in understanding real world networks. The properties include, for instance:

- The majority links of a node are contributed by nodes of its own natural community.

  This means that the major activities of an individual are within its own community.

- The degree of a node, v say, contributed by a community other than its own community is bounded by a constant.

  This means that an individual is unlikely to have many contacts with an external community.

- The length of degrees of a node is small, meaning that a node has links with only a small number of external communities.

This means that an individual is unlikely to contact nodes in many external communities.

- A seed node, interpreted as the hub of its own community, has a large number of neighbors from its own community.

  This means that a community has a hub, which leads its own community.

We now turn to prove Theorem 5.

(Proof of Theorem 5) For (1) and (2). For every node v, by the construction of G, with probability $1 - o(1)$, the degree of v contributed by a community other than v's own community is at most 1, and the degree of v contributed by nodes of its own community is at least $d > 1$. Both (1) and (2) follow.

For (3). By Theorem 1, the global edges associated to each homochromatic set is bounded by $O(\log \log n)$, which is also an upper bound of the length of degrees of a node.

If $a > 1$, then by the proof of Theorem 4(3), we know that with probability $1 - o(1)$, every homochromatic set with $t_S \in [T_2, T_4]$ has the number of global edges $O(\log^{1+\epsilon} n \log \log n)$. Since ϵ can be arbitrarily small, this upper bound is in fact $O(\log^{1+\epsilon} n)$ which is also an upper bound of the length of degrees of a node in S. Since there are $1 - o(1)$ fraction of nodes that are belonging to such S. (3) follows.

For (4). By the proof of Theorem 4(1), we know that if $t_S \leq T_3$, then with probability

$1-o(1)$, the size of S is $\Omega(\log^{a/2} n)$. Since almost all homochromatic sets are created no later than T_3, we only have to show that the first degree of the seed node v of such an S is at least $\Omega(\log^{\gamma} n)$. Note that a homochromatic set is constructed by preferential attachment scheme, and so is a small PA graph with few global edges. Since the degree of the first node of a PA graph is lower bounded by square root of the number of nodes, the first node has degree at least $\Omega(\log^{a/4} n)$. (4) holds.

This completes the proof of Theorem 5.

Theorem 5 explores the rules and patterns of both internal and external links of a node in a natural community structure of the network.

Similarly, we can investigate the rules and patterns of both internal and external links of a community in a natural community structure of a network.

Widths principle

In this section, we consider the rule and patterns of links of a community in a natural community structure of a homophyly network.

Definition 5 *Let $G = (V, E)$ be a homophyly network, and X be a homochromatic set (referred to as natural community) of G. We define the width of X in G to be the number of nodes x's such that $x \in X$ and $l(x) > 1$. We use $w^G(X)$ to denote the width of X in G.*

Intuitively, $w^G(X)$ is the number of nodes in X having external links to nodes outside of their own community.

Theorem 6 *(Widths Principle) Let $G = (V, E)$ be a network generated from $\mathcal{H}(n, a, d)$, where $a > 0$. Then the following properties hold:*

(1) For each homochromatic set S with $t_S \geq T_2$, the width of S is expected to be $O(\log \log n)$.

(2) If $a > 1$, then with probability $1 - o(1)$, for each homochromatic set S with $t_S \geq T_2$, the width of S is $O(\log^{1+\epsilon} n)$ for arbitrarily given constant ϵ.

By Theorem 6, a natural community has only a few nodes that have links with nodes outside of their own community. This intuitively reflects the reality that in human society, for a natural community, a small town say, there are only a few people having external links with people outside of their own town.

(Proof of Theorem 6) Note that the number of global edges of a homochromatic set S is an upper bound of the width of S. Thus (1) follows immediately from Theorem 1, and (2) follows from the fact (shown in the proof of Theorem 4(3)) that with probability $1 - o(n^{-1})$, every homochromatic set S with $t_S \geq T_2$ has at most $2a(\log^{1+\epsilon} n)(\log \log n)$ global edges for arbitrarily given constant ϵ and sufficiently large n. Theorem 6 holds.

The width of a community X determines the patterns of links from nodes in the community to nodes outside of the community.

Theorems 5 and 6 provide an insight to analyze the rules and patterns of both internal and external links of a node and a community in a natural community structure of a network. The significance of the theorems is the theoretical approach provided here. However, we don't think the exact bounds proved in the theorems fit well the real world networks. Nevertheless, we believe that extensions and modifications of our homophyly/kinship model may provide better fitness for the analyses and bounds of real world networks.

Finally, we notice that the existence of a small number of seed nodes is one of the most remarkable features of our homophyly networks. What is the principle that the remarkable seed nodes of a homophyly network must obey?

King node principle

In this section, we investigate the degrees of the seed nodes of a network of our homophyly/kinship model. Let $G = (V, E)$ be a homophyly network. For every natural community X of G, i.e., a homochromatic set of V, there is a unique seed node x_0 in X. By the construction, at the step at which x_0 is created, the degree of x_0 is d. Let x_1 be the second node in X. Then at the end of the step at which x_1 is created, the degree of x_0 is at least $2d$, and the degree of x_1 is d. By the construction of G, whenever a new element x is created as a member of X, x links to the existing nodes in X chosen with probability proportional to the degrees of the nodes of the same color. Intuitively speaking, the seed node of a community is similar to the queen bee of a colony of honey bees, so that the honey bees always follow their queen bee. This procedure implies that

the degree of x_0 maybe significantly larger than that of x_1 and every non-seed node of X during the construction of G. We will show that this intuition is true and can be mathematically proved. We also notice that the same theorem can be built for networks of the classic PA model, provided that at the beginning, the degree of the first node is twice of the degree of the second node.

Precisely, we have:

Theorem 7 *(King node principle) For $a > 0$, $d \geq 1$, let $G = (V, E)$ be a homophyly network generated from $\mathcal{H}(n, a, d)$. For every homochromatic set S, let v_0 be its seed node and v be its second node. Let d_{v_0} and d_v be the degrees of v_0 and v in G, respectively. Define function*

$$p(d) = \frac{2}{3 \cdot 2^{3d}} \cdot \binom{3d}{d} \cdot \prod_{t=1}^{2d} \frac{2t}{2t-1}.$$

Then:

(1) With probability at least $1 - p(d)$, the degree of v_0 is larger than that of v at each time step.

(2) For any $0 < \epsilon < 2$,

$$\Pr[d_{v_0} > (2-\epsilon)d_v] \geq 1 - \exp\left(-\frac{\epsilon^2 d}{6(3-\epsilon)^2}\right).$$

In Theorem 7, $p(1) = 2/3$, $p(2) = 4/7$, $p(3) = 16/33 \ldots$. Note that by Stirling's formula,

$$\binom{3d}{d} = O\left(\frac{1}{\sqrt{d}}\left(\frac{27}{4}\right)^d\right),$$

and

$$\prod_{t=1}^{2d} \frac{2t}{2t-1} = O(\sqrt{d}).$$

We have

$$p(d) = O\left(\left(\frac{27}{32}\right)^d\right),$$

which goes to 0 exponentially as d increases.

By Theorem 7 (1), if d is large enough, then the degree of v_0 is always larger than that of v with probability extremely close to 1 (0.99 say). By Theorem 7 (2), for sufficiently large (but still constant) d, with probability extremely close to 1 (0.99 say), the degree of v_0 in G is at least almost twice (1.99 say) of that of v in G.

Now we turn to prove the theorem.

(Proof of Theorem 7) At the beginning of this proof, let us clarify the evolution of connections of v_0 and v with other nodes. Suppose that v_0 and v are created at step t_0 and t_1 respectively. By the construction of S, at the step at which v_0 is created, its degree is d, which is contributed by the d global edges. At the step at which v is created, it creates d local edges between v and v_0. At the end of step t_1, the degree of v_0 reaches at least $2d$, and the degree of v is exactly d.

For every $t > t_1$, if a seed node x is created at step t, then for each of the d edges from x, the probability that the edge links v_0 is at least twice of that of the edge links v. If x is a non-seed node with the same color as v_0, then the expectation of the incremental degrees of v_0 is at least twice of that of v. If x is a non-seed node with color different from that of v_0, then the degrees of v_0 and v keep unchanged.

We consider all the time steps at which there is an edge connecting v_0 or v. We re-index these steps consecutively from 0, such that once an edge connects v_0 or v, the step increases by $+1$. We use $d_{v_0}(i)$ and $d_v(i)$ to denote the degrees of v_0 and v at the end of step i, respectively. Then we have that $d_{v_0}(0) \geq 2d$ and $d_v(0) = d$. From now on, we assume the worst case that $d_{v_0}(0) = 2d$.

At step i, $d_{v_0}(i) + d_v(i) = i + 3d$. Since the probability that an edge connects one of v_0 and v is proportional to their degrees, we have that $d_{v_0}(i+1) = d_{v_0}(i) + \Delta d_{v_0}(i)$ and $d_v(i+1) = d_v(i) + \Delta d_v(i)$, where $\Pr[\Delta d_{v_0}(i) = 0] = \Pr[\Delta d_v(i) = 1] = d_v(i)/(i+3d)$ and $\Pr[\Delta d_{v_0}(i) = 0] = \Pr[\Delta d_v(i) = 0] = d_{v_0}(i)/(i+3d)$.

For (1). It suffices to show that, with probability at least $1 - p(d)$, $d_{v_0}(i) > d_v(i)$ holds for every i. To prove the result, we first interpret our problem by a one-dimensional random walk. Consider a one-dimensional random walk on a line indexed by integers. For $i = 0, 1, 2, \ldots$, we use X_i to denote the location of a marker M at step i, which represents the difference of the degree of v_0 and the degree v and v_0 at step i.

The starting point X_0 is at least d ($d \geq 1$), which represents the fact that, at step 0, $d_{v_0}(0) -$

$d_v(0) \geq d$. For each i, M is moved one step to the left (in negative direction), which means d_v increases by 1, with probability $d_v(i)/(i+3d)$, and otherwise to the right (in positive direction), which means d_{v_0} increases by 1. Hence, $X_{i+1} = X_i + Z_i$ where $\Pr[Z_i = -1] = d_v(i)/(i+3d)$ and $\Pr[Z_i = 1] = d_{v_0}(i)/(i+3d)$. If $X_i = 0$ at some step i, it means that the degrees of v_0 and v become the same, then the random walk aborts.

The task of our proof is to show that the probability that d_v will ever catch up with d_{v_0} in infinitely many steps is upper bounded by $p(d)$. This is equivalent to the event that: $X_i = d_{v_0}(i) - d_v(i) > 0$ for all i's holds with probability at least $1 - p(d)$.

Note that $X_t = 0$ for some t only if $t \geq d$ and $t - d$ is even. Consider the paths of length $2k + d$ for integer $k \geq 0$. A key observation is that, for any fixed k, the probability for all paths of length $2k + d$ by which M is moved to the origin are equal. Formally, let

$$K_1(k) = \prod_{i=0}^{k+d-1} (i+d) \cdot \prod_{i=0}^{k-1} (i+2d),$$

and

$$K_2(k) = \prod_{i=0}^{2k+d-1} (i+3d).$$

Then this probability is $K_1(k)/K_2(k)$.

The reason is as follows. For a particular path P, let I_{left} be the set of steps at which M is moved to the left and I_{right} be those at which M is moved to the right. Since $X_{2k+d} = 0$ means that $d_{v_0}(2k+d) = d_v(2k+d) = k + 2d$, we have $|I_{\text{left}}| = k + d$ and $|I_{\text{right}}| = k$. Since at step i, M is moved to the left with probability $d_v(i)/(i+3d)$ and to the right with probability $d_{v_0}(i)/(i+3d)$

respectively, the probability that M follows path P is

$$\prod_{i \in I_{\text{left}}} \frac{d_v(i)}{(i+3d)} \cdot \prod_{i \in I_{\text{right}}} \frac{d_{v_0}(i)}{(i+3d)}.$$

Since the degrees of v and v_0 increase from d and $2d$ to $k+2d$, respectively, we have that

$$\prod_{i \in I_{\text{left}}} d_v(i) \cdot \prod_{i \in I_{\text{right}}} d_{v_0}(i) = K_1(k),$$

and

$$\prod_{i \in I_{\text{left}}} (i+3d) \cdot \prod_{i \in I_{\text{right}}} (i+3d) = \prod_{i \in I_{\text{left}} \cup I_{\text{right}}} (i+3d) = K_2(k).$$

Thus the probability of M following any particular path to the origin is exactly $K_1(k)/K_2(k)$.

Let N_{2k+d} denote the number of paths of length $2k+d$ by which M is moved to the origin for the first time, and p denote the probability that $X_i = 0$ for some $i \geq d$. We have

$$\begin{aligned}
p &\leq \sum_{k \geq 0} \frac{K_1(k)}{K_2(k)} \cdot N_{2k+d} \\
&= \frac{(3d-1)!}{(d-1)!(2d-1)!} \cdot \sum_{k \geq 0} \frac{((k+2d-1)!)^2}{(2k+4d-1)!} \cdot N_{2k+d} \\
&= \frac{(3d-1)!}{(d-1)!(2d-1)!} \cdot \sum_{k \geq 0} \frac{(2k+4d)}{(k+2d)^2 \cdot 2^{k+2d}} \cdot \frac{(k+2d)!}{(2k+4d-1)!!} \cdot N_{2k+d} \\
&= \frac{(3d-1)!}{(d-1)!(2d-1)!} \cdot \sum_{k \geq 0} \left[\frac{2k+4d}{(k+2d)^2} \cdot \frac{N_{2k+d}}{2^{k+2d}} \cdot \prod_{t=1}^{k+2d} \frac{t}{2t-1} \right] \\
&= \frac{(3d-1)!}{2^{3d}(d-1)!(2d-1)!} \cdot \sum_{k \geq 0} \left[\frac{N_{2k+d}}{2^{2k+d}} \cdot \left(\frac{2k+4d}{(k+2d)^2} \cdot \prod_{t=1}^{k+2d} \frac{2t}{2t-1} \right) \right].
\end{aligned}$$

Let $A(d) = \frac{(3d-1)!}{2^{3d}(d-1)!(2d-1)!}$ and $f(k) = \frac{2k+4d}{(k+2d)^2} \cdot \prod_{t=1}^{k+2d} \frac{2t}{2t-1}$. Then

$$p = A(d) \cdot \sum_{k \geq 0} \left(\frac{N_{2k+d}}{2^{2k+d}} \cdot f(k) \right).$$

Note that $\sum_{k\geq 0}\frac{N_{2k+d}}{2^{2k+d}}$ is the probability that an unbiased one-dimensional random walk starting from d will hit the origin in infinitely many steps. This form is exactly the sum over $k \geq 0$ of the probabilities that the marker reaches the origin for the first time at step $2k+d$. This probability is obviously at most 1. In fact, it equals 1 because of the folklore that the unbiased one-dimensional random walk starting from any fixed point will reach the origin in infinitely many steps with probability one. Moreover, since $f(k+1)/f(k) < 1$ for each $k \geq 0$, which means that $f(k)$ is a decreasing function of k, we have

$$p \leq A(d)\cdot f(0) = \binom{3d}{d}\cdot\frac{2d\cdot d}{2^{3d}\cdot 3d}\cdot\frac{1}{2}\cdot\prod_{t=1}^{2d}\frac{2t}{2t-1} = p(d).$$

(1) follows.

For (2). Let $f_i = X_i/V_i$ which denotes the fraction of v_0's degree. Since $E(f_i|f_{i-1}) = (X_{i-1}+f_{i-1})/V_i = f_{i-1}$, we know that $f_0, f_1, \ldots, f_r$ forms a martingale, where $f_0 = 2/3$ and r is the total number of time steps after re-indexing. Note that $|X_i - X_{i-1}| \leq 1$, then $|f_i - f_{i-1}| \leq 1/V_i$. Let $\mathbf{c} = (c_1, c_2, \ldots, c_r)$ where $c_i = 1/V_i$. Then the martingale $f_0, f_1, \ldots, f_r$ is **c**-Lipschitz. Thus, by Azuma's inequality in Lemma 2, since

$$\sum_{i=1}^{r} c_i^2 \leq \frac{1}{V_0} = \frac{1}{3d},$$

we have

$$\begin{aligned}
\Pr[d_{v_0} \leq (2-\epsilon)d_v] &= \Pr\left[f_r \leq \frac{2-\epsilon}{3-\epsilon}\right] \\
&= \Pr\left[f_r - f_0 \leq -\frac{\epsilon}{3(3-\epsilon)}\right] \\
&\leq \exp\left(-\frac{\epsilon^2}{9(3-\epsilon)^2} \cdot \frac{1}{2\sum_{i=1}^{r} c_i^2}\right) \\
&\leq \exp\left(-\frac{\epsilon^2 d}{6(3-\epsilon)^2}\right).
\end{aligned}$$

So $\Pr[d_{v_0} > (2-\epsilon)d_v] \geq 1 - \exp\left(-\frac{\epsilon^2 d}{6(3-\epsilon)^2}\right)$.

(2) follows.

This completes the proof of Theorem 7.

Theorem 7 explores the special property of the seed nodes in the homophyly networks. The property may play essential role in global behaviors of the networks.

Finally we remark that in the homophyly/kinship model, a natural community has a unique seed node. This is for simplicity for us to develop our theory. Of course, we assume that a natural community has a seed or hub, similar to many real world networking systems. It could be possible for a community to have a few seed nodes, in which case, the merging of the few seed nodes plays the same role as our seed node in the community. However, in real applications, if a community has a few seed nodes that compete among each other, then there will be new issues to resolve. For some applications, we may prefer to merge the few seed nodes into a strong base node of the community, for example, in network security and network system management etc. Nevertheless,

the uniqueness of seed node of a community is never a problem for either theory or applications of networks.

Evolutionary Games

Given a network $G = (V, E)$, the evolutionary games on G start with the following initial step: For every node $v \in V$, define the strategy of v at step 1, denoted by $s(v)[1]$, to be C with probability ϵ, and D, otherwise.

At step t, suppose that the strategy of v at step t, denoted by $s(v)[t]$ are defined for all v's. Then we use $P(v)[t]$ to denote the total payoffs of v obtained from the games with all its neighbors during step t. Then every node v randomly and uniformly chooses one of its neighbors as its reference node. Let u be the chosen reference node of v during step t, then define the strategy of v at step $t + 1$ by the Fermi rule. That is, the probability that node v adopts the last strategy of node u is defined by

$$P = \frac{1}{1 + \exp[-(P(u)[t] - P(v)[t])/T]}, \tag{19}$$

where T is a parameter representing the noise of the updating strategy. In all of our experiments, we set $T = 0.04$.

Given a model of networks, $\mathcal{M}$ say, and a type, we generate N networks of the model with

the same type. Suppose that $G_1, G_2, \cdots, G_N$ are the networks constructed by this way. For each network G_i, we implement M evolutions, denoted by $\mathcal{E}_1^{(i)}, \mathcal{E}_2^{(i)}, \cdots, \mathcal{E}_M^{(i)}$. Each evolution $\mathcal{E}_j^{(i)}$ on G_i proceeds as above. Then the evolutionary prisoner's dilemma games on G_i execute $3,000$ steps. For each round t, let $\theta_{i,j}(C)[t]$ be the fraction of nodes which have strategy C during step t. We define

$$\theta_{i,j}(C) = \frac{1}{1,000} \sum_{t=2,001}^{3,000} \theta_{i,j}(C)[t].$$

Define

$$\theta_i(C) = \frac{1}{M} \sum_{j=1}^{M} \theta_{i,j}(C).$$

And define

$$\theta(C) = \frac{1}{N} \sum_{i=1}^{N} \theta_i(C).$$

Then $\theta(C)$ is the average equilibrium of cooperators of networks of model $\mathcal{M}$ of the given type.

Gene Map of Classification

Fix a gene expression graph $G = (V, E)$ defined in the last section.

Suppose that $g_1, g_2, \cdots, g_N$ are all the genes. For an given gene g, we use $g(v)$ to denote the gene expression level of g for cell $v \in V$. By normalizing, we assume that the expectation of $g(v)$ for v is 0, and all the values $g(v)$ are real numbers in $[-1, 1]$.

Suppose that $X_1, X_2, \cdots, X_L$ form a partition of V. For a set X_i, we use $g(X_i)$ to be the average of $g(x)$ for all $x \in X_i$. For a gene g, we use X_g to denote the set X_i such that $g(X_i) = \max_j \{g(X_j)\}$.

For every j, we define B_j to be the set of all genes g's such that $X_g = X_j$, listed by the ordering of decreasing of the gene expression value $g(X_j)$.

Then a gene expression spectral of G by the classification $\{X_1, X_2, \cdots, X_L\}$ is a matrix of color codes of $L \times L$ blocks. In the matrix, the j-th row is the set B_j with ordering defined as above, and the j-th column is the set X_j listed with some fixed ordering. All the genes are listed by the ordering $B_1, B_2, \cdots, B_L$, in which each B_j has its own ordering by the gene expression profiles.

The gene expression spectral will explore the cell types and subtypes of tumors of cancers, and the set or block of genes that express the corresponding cell types and subtypes.

Data for the Cell Types of Normal Tissues

In Table 1, we give the similarities of the true types of the normal tissues found by our algorithm $\mathcal{E}^2$.

Our algorithm $\mathcal{E}^2$ finds 13 communities of the gene expression network of the normal tissues.

They are:

Community 1: Breast_BR_1, Breast_BR_2, Breast_BR_3, Breast_BR_4, Breast_93_I_184

Community 2: Prostate_PR_2, Prostate_PR_5, Prostate_95_I_255, Prostate_PR_6, Prostate_NLP10N, Prostate_NLP1N, Prostate_PR_3, Prostate_PR_7, Prostate_PR_4

Community 3: Lung_LU_1, Lung_LU_2, Lung_LU_7, Lung_LU_6, Lung_LU_3

Community 4: Lung_93_I_028_(I), Lung_HCTN_LUN2_ (18763_A2A), Kidney_KT_5, Kidney_Norm_613NPS, Kidney_Norm_613NS

Community 5: Colon_CR_1, Colon_CR_4, Colon_9912c071_CC, Colon_CR_5, Colon_CR_6, Colon_CR_9, Colon_CR_8, Colon_CR_10, Colon_CR_3,

Community 6: Colon_CR_2, Colon_CR_7, Uterus_UT_1, Uterus_UT_5

Community 7: Germinal_Center_GC2, Germinal_Center_GC3, Germinal_Center_GC45, Ger-

minal_Center_GC48, Germinal_Center_GC44, Germinal_Center_GC38

Community 8: Bladder_BL_1, Bladder_BL_4, Bladder_BL_2, Bladder_95_I_285, Whole_Brain_BRAIN_3, Bladder_BL_3, Uterus_UT_2, Uterus_UT_3, Uterus_UT_8

Community 9: Bladder_BL_5, Bladder_BL_6, Pancreas_PAN_1, Pancreas_PAN_2, Pancreas_Pan_8N, Pancreas_Pan_14N, Pancreas_Pan_43N, Pancreas_Pan_42N, Pancreas_Pan_41N, Pancreas_Pan_40N, Pancreas_Pan_11N, Pancreas_Pan13N

Community 10: Uterus_UT_4, Ovary_OV_1, Ovary_OV_2, Ovary_OV_4, Ovary_OV_3

Community 11: Peripheral_Blood_Poly_POLY1, Peripheral_Blood _Mono_MONO5, Peripheral_Blood_Mono_MONO6, Peripheral_Blood_Poly_POLY2, Peripheral_Blood _Poly_POLY3

Community 12: Kidney_KT_1, Kidney_Norm_622NO, Kidney_Norm_627, Kidney_KT_6, Kidney_KT_4, Kidney_KT_2, Kidney_KT_3, Kidney_Norm_613NO, Kidney_Norm_627NS

Community 13: Whole_Brain_BRAIN_1, Cerebellum_Ncer_NCB1, Cerebellum_Brain_Ncer_S-51, Cerebellum_Brain_Ncer_S-125, Whole_Brain_BRAIN_4, Whole_Brain_BRAIN_5, Whole_Brain_BRAIN_2

References

1. Chernoff, H. A note on an inequality involving the normal distribution. *The Annals of probability*, **9**, 533-535, (1981).

2. F. Chung and L. Lu. Complex graphs and networks. *American Mathematical Society*, ISBN-13: 978-0-8218-3657-6, (2006).

3. Barabási, A. L. & Albert, R. Emergence of scaling in random networks. *Science*, **286**(5439):509–512, (1999).

4. Kleinberg, J. Navigation in a small world, *Nature*, **406**, 845 (2000).

5. Bollobás, B. and Riordan, O. The diameter of a scale-free random graph, *Combinatorica*, **24** (4), 5 - 34, (2004).

6. Pittel, B. Note on the heights of random recursive trees and random m-ary search trees. *Random Structures and Algorithms*, **5**, 337 - 347, (1994).

ID	1	2	3	4	5
Type	Breast	Prostate	Lung	Colon	Germinal
Similarity	1.0	1.0	0.845	0.905	1.0
ID	6	7	8	9	10
Type	Bladder	Uterus	Peripheral	Kidney	Pancreas
Similarity	0.630	0.480	1.0	0.866	0.913
ID	11	12	13		
Type	Ovary	Whole	Cerebellum		
Similarity	0.894	0.676	0.655		

Table 1: Similarity of true cell types of normal tissues found by our algorithm $\mathcal{E}^2$.
